# d-α-tocopheryl polyethylene glycol 1000 succinate surface scaffold polysarcosine based polymeric nanoparticles of enzalutamide for the treatment of colorectal cancer: In vitro, in vivo characterizations

**DOI:** 10.1016/j.heliyon.2024.e25172

**Published:** 2024-02-02

**Authors:** Disha Shah, Sankha Bhattacharya, Girdhari Lal Gupta, Ketan Vinayakrao Hatware, Arinjay Jain, Laxmi Manthalkar, Niraj Phatak, Putrevu Sreelaya

**Affiliations:** a Department of Pharmaceutics, School of Pharmacy & Technology Management, SVKM’S NMIMS Deemed-to-be University, Shirpur, Maharashtra 425405, India; b Department of Pharmacology, School of Pharmacy & Technology Management, SVKM’S NMIMS Deemed-to-be University, Shirpur, Maharashtra 425405, India; c School of Pharmacy, International Medical University (IMU), Jalan Jalil Perkasa 1, Bukit Jalil, 57700 Kuala Lumpur, Malaysia

**Keywords:** Enzalutamide, TPGS, Box-behnken design, HCT 116, DNA fragmentation assay, Cell cycle analysis

## Abstract

In this study, Enzalutamide (ENZ) loaded Poly Lactic-*co*-Glycolic Acid (PLGA) nanoparticles coated with polysarcosine and d-α-Tocopheryl polyethylene glycol 1000 succinate (TPGS) were prepared using a three-step modified nanoprecipitation method combined with self-assembly. A three-factor, three-level Box-Behnken design was implemented with Design-Expert® software to evaluate the impact of three independent variables on particle size, zeta potential, and percent entrapment efficiency through a numeric optimization approach. The results were corroborated with ANOVA analysis, regression equations, and response surface plots.

Field emission scanning electron microscopy and transmission electron microscope images revealed nanosized, spherical polymeric nanoparticles (NPs) with a size distribution ranging from 178.9 ± 2.3 to 212.8 ± 0.7 nm, a zeta potential of 12.6 ± 0.8 mV, and entrapment efficiency of 71.2 ± 0.7 %. The latter increased with higher polymer concentration. Increased polymer concentration and homogenization speed also enhanced drug entrapment efficiency. In vitro drug release was 85 ± 22.5 %, following the Higuchi model (R2 = 0.98) and Fickian diffusion (n < 0.5).

In vitro cytotoxicity assessments, including Mitochondrial Membrane Potential Estimation, Apoptosis analysis, cell cycle analysis, Reactive oxygen species estimation, Wound healing assay, DNA fragmentation assay, and IC50 evaluation with Sulforhodamine B assay, indicated low toxicity and high efficacy of polymeric nanoparticles compared to the drug alone. In vivo studies demonstrated biocompatibility and target specificity. The findings suggest that TPGS surface-scaffolded polysarcosine-based polymer nanoparticles of ENZ could be a promising and safe delivery system with sustained release for colorectal cancer treatment, yielding improved therapeutic outcomes.

## Highlights

• Enzalutamide (ENZ) scaffolded PLGA-polysarcosine (PSAR) nanoparticles were fabricated with outer coating of TPGS for solubility enhancement.
• ENZ-PLGA-PSAR-TPGS NPs characterized by SEM, TEM, DSC, XRD, FT-IR.
• In vitro anti-cancer cyto-toxicity evaluation performed for ENZ-PLGA-PSAR-TPGS NPs in HCT-116 cell lines.
• Haemolysis, Platelet aggregation, Acute toxicity conducted to analyze biocompatibility.
• Anti-Cancer activity of ENZ-PLGA-PSAR-TPGS NPs evaluated by Apoptosis, MMP, ROS, wound healing, DNA fragmentation studies.

## 1 Introduction

The second most prevalent cancer-related death in the US is colorectal cancer(CRC) [1]. Lung cancer is the primary cause of cancer death in both China and the USA, where it is more prevalent than breast cancer [2]. Based on incidence data from population-based cancer registries and death data from the National Centre for Health Statistics, the [3]. Incidence rates for CRC from 2012 to 2016 ranged from 30 (per 100,000 people) for Asian/Pacific Islanders to 45.7 (per 100,000 people) for Blacks, and 89 (per 100,000 people) for Alaska Natives [4]. The probability of suffering from CRC is about 4 %–5 % and the risk for developing CRC is associated with personal features or habits such as age, chronic disease history and lifestyle [5]. Nevertheless, intrinsic and acquired resistance of tumor cells, dose-limiting toxicities of platinum derivatives, and limited drug bioavailability are the major limitations of these treatments. The high poor prognosis, incidence and mortality of CRC indicated that elucidation of the underlying mechanism is an urgent unmet medical need, in order to improve diagnostic methods and therapies [6]. As per the national cancer institute predictions, in upcoming years, there would be 15 million new cases of cancer every year [7].

**Fig. 1:**
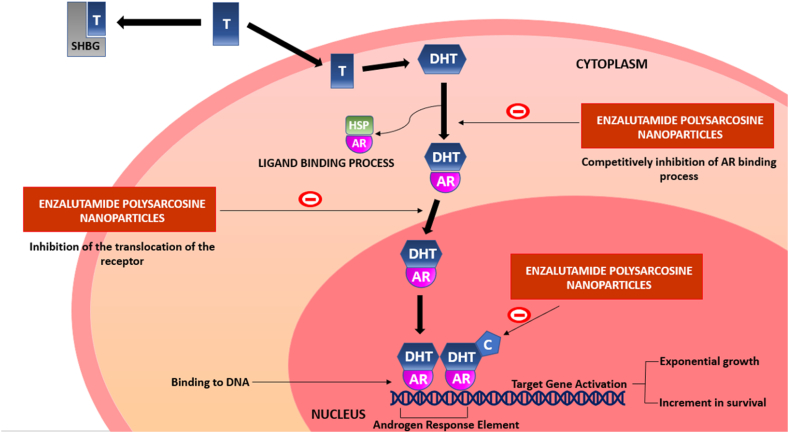
Mechanism of Enzalutamide inhibition w.r.t testosterone inside cell via three processes.

**Fig. 2:**
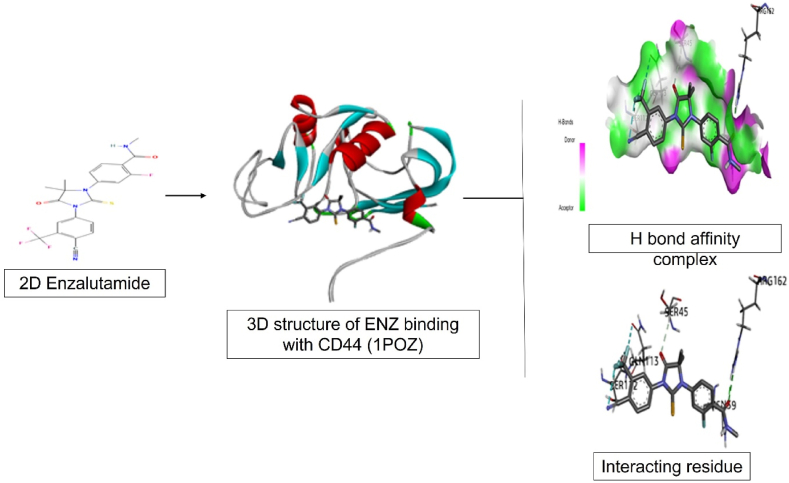
Molecular docking of CD44 antigen (1POZ) Complexed with Enzalutamide showing 3D model of the interactions and the 2D interaction patterns and H-bond interaction.

Enzalutamide is a second-generation androgen receptor (AR) inhibitor [8] which successfully extends the survival of patients with castration-resistant prostate cancer (CRPC) by an additional 4.8 months [9]. Enzalutamide has a stronger affinity for the androgen receptor than the first-generation anti-androgens flutamide and bicalutamide, leading to a better response in the context of castrate resistance [10]. In contrast to other antiandrogen medications now on the market, enzalutamide prevents DNA binding, coactivator recruitment, and androgen receptor nuclear translocation which is depicted in Fig. 1. Additionally, it causes tumor reduction in xenograft models, has a higher affinity for the receptor, and has no known agonistic effects [11]. This research includes repurposing of the drug ENZ for CRC. Farahmandlou N. et al. established correlation between the ENZ acting on testosterone with human CRC in *in vitro* mode by conducting cell viability via MTT assay. The results depicted that at 1000 μg/mL concentration showed significant decrease in cell viability in HT-29 cell line [12]. In order to confirm the hypothesis, we conducted in silico molecular docking of ENZ drug with CD44 receptors. Type I transmembrane glycoproteins belonging to the CD44 family are expressed on a wide range of cells, including those with epithelial, mesenchymal, and hematopoietic origins [13]. For the development and progression of tumours in CRC, CD44(1POZ) is crucial functionally [14]. CD44 is currently commonly employed in CRC as a cell surface marker of CRC-initiating cells [15]. Energy scoring helps in analysing affinity of ENZ towards CD44 receptor [16]. The energy score was found to be −4.66 in which the negative sign depicts bonding of ligand to receptor in a spontaneous mode without consuming energy [17]. Interacting residues that are proteins made up with amino acids mainly are ARG:162, ASN:39; SER:45, GLN:113 and SER:112 playing a big role in anti-cancerous activity which interact to form stable native structure [18]. The molecular docking depicted in Fig. 2 shows the interaction between ENZ ligand and CD44 receptor having activity on it predominantly present in CRC. Thus, to improve the drug resistance, increase bioavailability and increased therapeutic effectiveness, biocompatible nanoparticle (NP) drug delivery technology was adopted. In order to have a proper strength to form NPs, Poly lactic-*co*-glycolic acid (PLGA) was utilized having larger drug capacity [19]. Targeted nanoparticles are designed to increase diagnostic and therapeutic efficacy while reducing unproductive dispersion [20]. PLGA NPs are used to improve efficacy and reduce cytoxicity in CRC treatment [21]. Poly (ethylene glycol) (PEG) is used as polymer to provide enhanced pharmacokinetics but due to limitations that is non-degradability and immunogenicity [22], Polysarcosine (PSAR) is used [23] as it possesses characteristics of non-ionic polypeptoid is introduced as a polymer [24]. Despite being based on endogenous material, it has PEG-like characteristics such as good water solubility, protein resistance, low cellular toxicity, and a non-immunogenic nature. Bromoacetic acid and methylamine can be used to make sarcosine, which is then easily converted into its N-(thio) carboxyanhydride and polymerized under living conditions [25].

TPGS is also termed as D-alpha tocopherol polyethylene glycol succinate [26], is a water-soluble derivative of natural vitamin E that is created by esterifying vitamin E succinate with poly (ethylene glycol) (PEG) 1000 [27]. It can be functionalized as a permeation agent [26], emulsifier [28] and bioavailability enhancer of hydrophobic medicines due to its amphiphilic structure, which consists of a lipophilic alkyl tail and a hydrophilic polar head part [29]. It has been shown to induce apoptogenic action against a variety of cancer types when used as an anticancer drug [30]. It can target cancer cells present in mitochondria, causing mitochondrial destabilization and the activation of mitochondrial apoptotic mediators. They display nontoxicity to healthy cells and tissues while specifically inducing apoptosis in malignant cells [31]. Through modification of its intrinsic physicochemical features, the addition of TPGS could further enhance delivery in Multi-drug resistance (MDR) [32].TPGS bypasses resistance caused by P-glycoprotein and Cytochrome inhibitors [33]. Incorporation of TPGS has been proven to provide controlled drug release in some formulations as well. Thus, due to the presence of excellent properties of TPGS, it is scaffolded in nanoparticle. After that, TPGS conjugated ENZ self-assembled nanoparticulate system based on PLGA and polysarcosine was created [34]. ENZ is BCS class II drug [35], which depicts low solubility of the drug and thus TPGS coating enhances the solubility of the drug.

In this research, in order to optimize the formulation of NPs, Box-Behnken design is used to analyze the independent and dependent factors [36]. These experimental designs have perks in lower numbers of trials for optimization process [37],which is time saving process and provides specified data on influence of individual and combined effects of formulation and process response variables to obtain final optimized formulation [38]. Using this, ENZ-PLGA-PSAR-TPGS NPs are formulated in order to provide sustained release, increase bioavaibility via increasing the solubility and reduce toxicity. *In vitro* cyto-toxic studies were conducted in this research in order to analyze the effectivity of the ENZ-PLGA-PSAR-TPGS NPs w.r.t plain drug ENZ and analyze the toxicity. As a result, in order to reduce less target specificity and harmful effects in chemotherapy, ENZ-PLGA-PSAR-TPGS NPs can be used as a novel potent formulation in treating colorectal cancer with low toxicity and more bioavaibility in body.

## 2 Materials & methods

### 2.1 Materials

Enzalutamide was obtained as a gift sample from BDR Lifesciences Private Limited, Gujarat, India. The PLGA polymer (50/50) with an inherent viscosity of 0.95–1.20 dl/g was purchased from Nomisma Healthcare Private Limited, Gujarat, India. PSAR (Molecular weight: 89.09 g/mol; Melting point: 208–212 °C) was procured from Sigma-Aldrich Limited, Bengaluru, India. TPGS was sourced from Thermo Fisher Scientific India Private Limited, Mumbai, India. Methanol was purchased from Merck Limited, Mumbai, while acetone, Tween 80, and diethyl ether were obtained from Rankem, Mumbai. PVA was acquired from Research Lab, Mumbai. Dialysis tubing cellulose membranes (1 Dia 8/32, 6.3 mm; 12400Da) were purchased from Himedia company, India.

### 2.2 Methods

#### 2.2.1 Spectroscopical analysis of drug ENZ

Spectroscopy is a scientific technique related to the study of the absorption and emission of electromagnetic waves by matter. It provides valuable information about the interaction between particles, such as electrons, protons, and ions, within a given substance [39]. Spectroscopic analysis is commonly utilized in pharmaceutical and chemical research to assess various aspects of a substance, including drug formulations [40].

##### 2.2.1.1 Analysis by UV spectrophotometer

The stock solution for Enzalutamide (ENZ) was prepared as follows: A 100 μg/ml stock solution was created by dissolving 10 mg of ENZ drug in a 100 ml volumetric flask using methanol. Subsequently, this stock solution was further diluted with methanol in 10 ml aliquots to obtain concentrations of 2, 4, 6, 8, and 10 μg/ml, respectively.

For qualitative analysis, the solution was subjected to UV–vis spectroscopy to determine the maximum wavelength. This involved using a UV–vis spectrophotometer to measure the absorbance of the solution at different wavelengths of light. The wavelength at which the absorbance is highest represents the maximum wavelength, providing valuable information about the spectral characteristics of the solution [41]. The absorption spectra of the standard solution were scanned over a wavelength range of 200 nm–400 nm against a blank of distilled water to obtain the spectra. Subsequently, these dilutions were utilized to establish the calibration curve at a specific wavelength, in this case, at 236 nm. The observed absorbance values were plotted on an overlay display plot, facilitating the visualization and interpretation of the calibration curve. This curve provides a quantitative relationship between the concentration of the analyte (Enzalutamide) and its absorbance at the selected wavelength, enabling accurate concentration determinations in subsequent analyses.

##### 2.2.1.2 Estimation of ENZ by RP-HPLC

###### 2.2.1.2.1 Preparation of stock solution and ENZ

Approximately 10 mg of ENZ was taken into a 100 ml of volumetric flask. 20 mM Ammonium acetate: Acetonitrile (pH-4.6) at 60:40 ratio was taken as a mobile phase. The prepared mobile phase was degassed in ultrasonic water bath for 5 min.

###### 2.2.1.2.2 Chromatographic conditions

The chromatography system was of PerkinElmer, Series 200 consisting of well-equipped UV–visible detector. The flow rate of mobile phase including ammonium acetate buffer (pH = 4.6, 20 mM) and acetonitrile (60:40, v/v) was set at 1.0 ml/min [42]. The absorption wavelength for the assay was set at 236 nm throughout the 10-min run. Chromatographic separation was achieved under isocratic elution with C′18 column (5 μm, 4.6 * 100 mm; Kromasil) protected by a guard column of the same phase.

This helped in determining the purity of the drug ENZ and acts as pre-formulation study.

#### 2.2.2 Experiment design for optimization

When experimental variables and outcomes exhibit a nonlinear relationship, the Box-Behnken design emerges as a powerful tool for optimization. This design excels at deciphering nonlinear quadratic effects and interactions between variables, providing a comprehensive understanding of complex systems. In this study, Design-Expert 13.0 software guided a three-factor, three-level Box-Behnken experimental design, leading to 17 carefully crafted runs intended to optimize polymeric nanoparticle formulation. The independent variables under investigation included polysarcosine concentration, PLGA concentration, and homogenization speed [42]. Within this framework, particle size, zeta potential, and entrapment efficiency served as dependent variables, also known as response variables. Each independent variable was meticulously examined across three levels—high, medium, and low—to capture a comprehensive response profile. The specific values employed for each variable are meticulously detailed in Table 1.

**Table 1:** Three Levels of independent and dependent variables in the experimental design.

PARAMETER	UNITS	LOW (−1)	HIGH (+1)
X1- PSAR concentration	%w/v	0.2	0.6
X2- PLGA concentration	%w/v	1.5	2
X3- Homogenization speed	rpm	13000	14000
DEPENDENT VARIABLES		LOW	HIGH
Y1- Particle size		178.9	212.8
Y2- Zeta Potential		8.4	13.1
Y3- % Entrapment efficiency		40.3	75.4

These values were analyzed based upon initial trial experiments as well as review of available literature. Design matrix had an inclusion of 14 factorial and 3 centre points with a specified set of 17 formulations. The optimization of X1, X2, X3 was kept in check and was aimed in order to minimize size of particle along with stability of Zeta potential and maximum entrapment.

The entire set of formulations was prepared using a randomised selection process, eliminating any chance of bias. Equation shown below provides the expression produced by the Box Behnken nonlinear quadratic model that is

R_i_ = b_0_+ R_1_X_1_ + R_2_X_2_+ R_3_X_3_ + R_12_ × 1 × _2_ + R_13_ × 1 × _3_ + R_23_ × 2 × _3_ + R_11_X_1_^2^+ R_22_X_2_^2^+ R_33_X_3_^2^

In this equation, R denotes the measured response at each level of the independent variables within the study design. X1, X2, and X3 represent the influencing factors, while b0 signifies the intercept. R1 through R33 embody the regression coefficients corresponding to each variable and their interaction terms, meticulously calculated from the experimental data. The interactions between variables are captured by X1X2, X1X3, and X2X3, while X12, X22, and X32 depict the quadratic terms within the equation.

#### 2.2.3 Preparation of ENZ-PLGA NPs

ENZ-loaded PLGA NPs were synthesized by a modified nano precipitation method and oil in water (o/w) method. In this experiment, 10 mg of enzyme were carefully combined with a tailored PLGA solution. This solution, containing 1.5–2 % PLGA dissolved in 10 ml acetone, formed the organic phase. A separate aqueous phase was prepared by dissolving 2 drops of Tween 80 in 15 ml water. With meticulous precision, the organic phase was gradually added drop-wise to the aqueous phase using a syringe. Following overnight stirring at room temperature, the organic solvent gently evaporated, prompting the PLGA molecules to self-assemble around the enzyme, resulting in the formation of enzyme-encapsulated PLGA nanoparticles. This technique provides a powerful platform for enzyme protection and controlled release, holding significant potential for future applications [43]. A placebo consisting of drug-free nanoparticles was synthesized by replicating the preparation process, excluding the drug itself.

#### 2.2.4 Prime-coating of polysarcosine on ENZ-PLGA NPs

A novel method was employed to synthesize polysarcosine-coated ENZ-PLGA nanoparticles (ENZ-PLGA-PSAR NPs). The process began with the dropwise addition of 40 ml methanol to a pre-existing ENZ-PLGA NPs solution under constant stirring. Subsequently, (0.2–0.6 %w/v) PSAR was incorporated and the mixture stirred overnight at room temperature. The addition of 50 ml diethyl ether solution temporarily induced a faint pink coloration. To enhance stability and viscosity, 2.5 % PVA (pre-treated along with 5 ml nitric acid) was introduced to ENZ-PLGA-PSAR solution. Finally, mixture was homogenized at 13000–14000 rpm using a homogenizer and sonicated using an ultrasonic processor [44].

#### 2.2.5 Coating of TPGS over ENZ-PLGA-PSAR NPs

TPGS was taken in 0.5 ml quantity and was dissolved in 3 ml methanol and was poured in ENZ-PLGA-PSAR NPs under stirring using REMI magnetic stirrer at normal temperature of room. Following overnight stirring, the ENZ-PLGA-PSAR-TPGS NPs solution underwent centrifugation. The supernatant was discarded, and the pellet was resuspended in ultrapure water to restore the original volume of the particle suspension [45].

#### 2.2.6 Optimization of the formulation

Data on several dependent variables were provided via the computer-assisted optimization process using the Design-Expert® programme, which then employed analysis of statistics to produce the optimized formulation with projected values of various structural components such as concentration of polymers and homogenization speed. Particle size, zeta potential, and entrapment efficiency results of dependent variables were likewise predicted by the procedure. In order to validate the model, the findings for three times the above-mentioned data were replicated to create the optimized formulation.

#### 2.2.7 Characterization of PLGA-PSAR-TPGS-NPs

##### 2.2.7.1 1: size of particle and zeta potential

A Malvern Zetasizer Version 7.11 (Malvern Instruments Ltd, UK) served as the instrument for characterizing the prepared formulations, with particle size determined through dynamic light scattering and zeta potential assessed by measuring the electrophoretic mobility of the nanoparticles under an applied electric field [46]. Milli-Q water was employed as the diluent for all samples in preparation for analysis. We report both the average particle size and the polydispersity index (PDI), a dimensionless metric that captures the extent of size distribution variation. Lower PDI values correspond to a more homogenous particle population.

##### 2.2.7.2 Morphological analysis

The scanning electron microscopy of optimized PLGA-PSAR-TPGS-NP was performed by JSM-IT800 Field Emission Scanning Electron Microscope (FESEM) (Tokyo, Japan). Samples were fixed and sputtered on metal plates with a gold–palladium combination at a thickness of 100 Å and observed at an elevated voltage of 20 kV [47]. To determine the morphology of the optimized ENZ-PLGA-PSAR-TPGS NP and for proper magnificent images, TEM was performed (Hitachi 7500, Tokyo, Japan). Before conducting TEM, the nanoparticles were coated with carbon and put in stained copper grid phosphotungstic acid (1 %) [48]. The digital micrograph-generated software interprets developed images from TEM studies. Morphological analysis was conducted in order to analyze the surface characteristics of the optimized polymeric NPs.

##### 2.2.7.3 Drug entrapment efficiency

The centrifugation method served as the technique for determining the entrapment efficiency of all DOE formulations, allowing us to measure the extent of drug encapsulation [49]. To quantify the amount of free drug in the formulation, the following steps were performed:

Centrifugation: The formulation was placed in an Eppendorf tube and centrifuged at 10,000 rpm for 30 min. This process separated the supernatant liquid, containing the free drug, from the remaining formulation components.

Filtration: The supernatant liquid was carefully collected and filtered to remove any particulate matter that could interfere with subsequent analysis.

Spectrophotometric analysis: The filtered supernatant was then subjected to UV–Visible spectrophotometry at a wavelength of 236 nm, a wavelength known to be sensitive to the specific drug being analyzed. The absorbance measurement at this wavelength was recorded.

Free drug concentration calculation: Using a previously established calibration curve, the absorbance value was translated into a corresponding free drug concentration.

Entrapment efficiency calculation:

With the free drug concentration determined, the entrapment efficiency (EE) was calculated using the following formula.

##### 2.2.7.4 Drug release (in-vitro) and kinetic studies

We utilized a dialysis tube diffusion system to characterize the in vitro release of drugs from the formulations. This system provides a controlled environment to study the rate and extent of drug release from the formulations, reflecting their potential drug delivery behaviour [50]. NPs has a specified content of ENZ present in itself which was furthermore suspended in solution of 1 mL of phosphate buffer saline (PBS pH 5.5) and is further added in dialysis tube (MW cutoff 10–12 kDa). Tube was inserted into 50 mL of PBS (pH 5.5) with both ends tightened. In order to provide the sink conditions, PBS quantifying to 0.5 mL was added. Formulated medium was heated up to 37 ± 0.5 °C and stirred continuously at speed of 200 rpm. At predefined intervals, a 5 mL sample was taken, and an equal volume of new medium was introduced to keep the final volume, or sink condition, throughout experiment. All test samples were analyzed in triplicate manner and results are expressed as mean ± SD (n = 3).

To elucidate the kinetics and mechanisms governing drug release, we employed regression analysis to fit the experimental drug release data to various mathematical models. This analysis yielded coefficient constants that shed light on the order of release and the underlying mechanisms involved. (See Supportive Information for further details) [51].

##### 2.2.7.5 Fourier transform infrared (FTIR) spectroscopy

In order to analyze any kind of potential physicochemical interactions between respective formulation components, usage of FTIR spectroscopic research was carried out [52]. Drug ENZ, ENZ-PLGA-formulation like PSAR-TPGS NPs, polymers like PLGA and PSAR, Placebo (drug-free formulation) and Physical mixture were determine with usage of PerkinElmer FTIR spectrometer at a resolution of 4 cm^−1^ in range of measurement of 4000 to 400 cm^−1^ [53]. In order to analyze and verify potential physicochemical interactions, the FTIR spectra of various components were compared with those of the ENZ-PLGA-PSAR-TPGS and PLGA-PSAR-TPGS NPs.

##### 2.2.7.6 Differential scanning calorimetry (DSC)

Analysis conducted by DSC served as the technique for characterizing the solid-state nature of ENZ in the optimized NP formulation, providing insights into its physical form and potential interactions with other formulation components [54]. Placebo, optimized drug formulation and pure drug ENZ were determined precisely with the usage of DSC. Determination of all these components were carried out on a system of DSC 3, procured from Mettler Toledo (Ohio, USA). Precisely, 100 mg quantity of sample was weighed and heated at temperature of from 25 to 250 °C maintaining rate of 10 °C/min in inert condition of nitrogen flowing at rate of 50 mL/min.

##### 2.2.7.7 X-ray diffraction (XRD)

XRD analysis was performed by Bruker D2 Phaser (X-ray diffractometer, Karlsruhe, Germany) for the pure drug ENZ, placebo i.e., PLGA-PSAR-TPGS NPs and optimized ENZ-PLGA-PSAR-TPGS NPs formulation. All the XRD patterns were recorder at ambient temperature at 2θ diffraction angle in a range of 5–50° in order to analyze the crystallinity form [55]. XRD analysis was conducted in order to analyze the crystallinity nature of the optimized NP.

#### 2.2.8 *In vitro* anticancer studies

##### 2.2.8.1 Sulforhodamine B (SRB) assay

Assessment of cytotoxicity for the given samples on the HCT116 cell line was conducted using the SRB Assay. This involved cultivating cells quantity of (8000 cells/well) in 96-well plate for 24 h in DMEM medium [56] which was continuously nourished with solution of 1 % antibiotic solution at 37 °C and 10 % FBS and 5 % CO_2_. These cells were treated in varied different concentrations dosage of 1–2500 μM on next day [57]. Various concentrations were prepared in an incomplete medium. Following a 24-h incubation period, 100 μL of Tri Chloro Acetic Acid (TCA, 10 %) was introduced to each well, and plate was incubated for an additional hour. Subsequently, the plate was washed with DM water and air-dried at room temperature. SRB Solution, with a final concentration of 0.04 %, was applied to each well and allowed to sit for 1 h. After this incubation period, the plate underwent washing with 1 % (v/v) acetic acid to eliminate unbound dye, followed by air-drying at room temperature. Tris base solution (pH = 10.5) was added to each well and shaken on an orbital shaker for 10 min to solubilize the protein-bound dye. The absorbance was measured using an Elisa plate reader (iMark, Biorad, USA) at 510 nm. Triplicate analyses were performed for all test samples, and the results are presented as mean ± SD (n = 3).

##### 2.2.8.2 Cell cycle analysis

HCT116 cells were seeded and exposed to the samples at the IC50 dose of the formulations for a duration of 24 h [58]. Following the incubation period, cells were harvested using trypsin and collected in a 1.5 ml tube. After a single wash with 500 μl of chilled PBS, approximately 1 x 10^6 cells were suspended in 100 μl of PBS. The cell suspension was gently vortexed to achieve a mono-dispersed cell suspension with minimal aggregation. Subsequently, the cells were fixed by transferring this suspension with a pipette into centrifuge tubes containing 900 μl of 70 % ethanol, placed on ice, and then incubated for a minimum of 2 h at 4 °C. The fixed cells could be stored in 70 % ethanol at 4 °C for several weeks.

After fixation, the cells were centrifuged, and the pellet was suspended in 500 μl of Propidium Iodide (PI) staining solution (0.1 % (v/v) Triton X-100, 10 μg/ml PI, and 100 μg/ml DNase-free RNaseA in PBS). This suspension was kept in the dark at room temperature for 30 min or at 37 °C for 10 min. The samples were then acquired on a flow cytometer. Triplicate analyses were conducted for all test samples, and the results are presented as mean ± SD (n = 3).

##### 2.2.8.3 Cellular apoptosis (flow cytometry)

HCT116 cell lines were seeded, cultured and subjected up to IC-50 dose of compound. Subsequent to treatment, cells underwent two washes with cold PBS. Resuspended cells were placed in 1X binding buffer at a concentration of 1x10^6 cells/ml, and the cells were divided into distinct groups, namely Unstained cells, Control group, Annexin only, PI only, and Treatment [59]. Following the division into labelled tubes, Annexin V FITC and PI were introduced to their respective tubes. After vortexing and an incubation period of 15 min at room temperature, 1X Binding buffer was added to each tube. Subsequently, the samples were analyzed by a Flow Cytometer within 1 h. Triplicate analyses were conducted for all test samples, and the results are presented as mean ± SD (n = 3).

##### 2.2.8.4 Reactive oxygen Spieces (estimation through flow cytometry)

HCT116 cells were cultivated appropriately and exposed up to IC-50 doses of samples, followed by a 24-h incubation period. Subsequent to the incubation, the medium was removed, and cells were harvested using trypsin EDTA. The harvested cells were then collected in a 1.5 ml tube and subjected to a single wash with 500 μl of chilled PBS [60]. Ultimately, the cell pellet was resuspended in 100 μl of PBS containing 2 μM DCFDA. The samples were then analyzed using a Flow Cytometer within a 1-h timeframe. All test samples underwent triplicate analyses, and the results are presented as mean ± SD (n = 3).

##### 2.2.8.5 Mitochondrial membrane potential estimation by flow cytometry

HCT116 cells were cultured and exposed to the IC50 dose of both the standard and formulations, followed by a 24-h incubation. After the incubation period, the medium was aspirated, and cells were harvested using trypsin EDTA. The harvested cells were collected in a 1.5 ml tube and subjected to a single wash with 500 μl of chilled PBS. Subsequently, the cell pellets were resuspended in 400 μl of JC1 staining buffer containing 2 μM JC-1 dye. The samples were then promptly acquired using a Flow Cytometer within a 1-h timeframe [61]. All test samples underwent triplicate analyses, and the results are presented as mean ± SD (n = 3).

##### 2.2.8.6 DNA fragmentation assay

HCT116 cells, seeded at a density of 10,000 cells per well, were cultured in a 96-well plate for 24 h in DMEM medium supplemented with 10 % FBS and 1 % antibiotic solution at 37 °C with 5 % CO2. On the following day, the cells were treated with the IC-50 dosage of the formulations and incubated for an additional 24 h. After this incubation period, the deceased cells were collected, and DNA was isolated using a standard DNA isolation protocol.

The isolated DNA samples were characterized through 1 % agarose gel electrophoresis, running for 30 min at 90V. Gel images were captured using a gel documentation system (CAMAG- Repostar 3 equipped with Canon 1300D). All test samples underwent triplicate analyses, and the results are presented as mean ± SD (n = 3).

##### 2.2.8.7 Wound healing assay

HCT116 cells, seeded at a density of 10,000 cells per well, were cultured in a 96-well plate for 24 h in DMEM medium supplemented with 10 % FBS and 1 % antibiotic solution at 37 °C with 5 % CO2. The following day, a scratch was made in the cell monolayer using a 200 μl tip, and the cells were subsequently treated with the specified concentration of the formulation. Photographs were taken at various time intervals ranging from 0 to 48 h.

The gap area resulting from the scratch was analyzed using Image J Software (NCBI) and was further presented in a graphical format to illustrate the changes over time [62]. Relative units underwent triplicate analyses, and the results are presented as mean ± SD (n = 3).

#### 2.2.9 in vivo studies

##### 2.2.9.1 Haemolysis study

Hemocompatibility analysis was performed to assess hemolysis following the administration of ENZ, PLGA-PSAR-TPGS NPs, and ENZ-PLGA-PSAR-TPGS NPs at concentrations of 10, 50, and 100 μg/mL. For negative control identification, Triton X-100 treatment was employed [63]. A 4 mL blood sample was drawn from male albino rats (220g) using a 23-gauge needle from the lateral saphenous vein of the animal (SPTM/2022/IAEC/18). The blood sample was then centrifuged at 1344×*g* for 10 min, and the plasma layer was carefully removed. The erythrocytes were washed three times with saline water and incubated with a 100 μg/mL concentration of the samples at 37 °C. Samples of 200 μL each were collected at 0, 1–10 h and centrifuged at 1344×*g* for 10 min.

During the incubation, hemoglobin transformed into oxyhemoglobin. The obtained erythrocytes were observed under a microscope, and absorbance readings were taken at 570 nm for different concentrations of samples. All test samples were analyzed in triplicate, and the results are expressed as mean ± SD (n = 3). The percentage of hemolysis can be calculated using the following equation.

##### 2.2.9.2 Platelet aggregation study

A 1 mL blood sample was collected in heparinized tubes to prevent coagulation. Subsequently, the blood was incubated with ENZ, PLGA-PSAR-TPGS NPs, and ENZ-PLGA-PSAR-TPGS NPs for a duration of 2 h. The platelet aggregation was then examined both quantitatively and qualitatively [64]. The qualitative analysis was conducted through microscopic examination of stained blood smears. Blood smears were prepared on clean glass slides after the 2-h incubation period. The slides were air-dried and subsequently stained with Leishman's stain for 5 min (Span Diagnostics, India). Following the staining, the slides were rinsed with distilled water, and a cover glass was placed on each slide for visualization under an optical microscope using an immersion objective. Images were captured using a digital camera.

As a control, whole blood incubated with an equivalent volume of PBS was used as the spontaneous control. All test samples underwent triplicate analyses, and the results are expressed as mean ± SD (n = 3).

#### 2.2.10 Acute toxicity

To assess the safety and compatibility of the components in the produced polymeric NP formulation, an acute toxicity study was conducted following the criteria outlined by the Organisation for Economic Co-operation and Development (OECD) standards. The primary objective of the study was to determine the toxicity of the developed system and its distribution throughout the body.

Six rats were included in each of the three groups, with the rats divided into two groups (test and control) as per protocol (SPTM/2022/IAEC/18). The test group, labelled as group II, received a single dose of ENZ-PLGA-PSAR-TPGS NPs, while the control group was designated as group I. Rats in both groups were housed separately in cages with clean ventilation, provided with healthy food and water.

Throughout a fourteen-day observation period, the animals were closely monitored for various physical characteristics, including signs of toxicity and mortality rates. On the fifteenth day, the rats were euthanized, and blood was drawn for hematological analysis. Additionally, internal organs such as the heart, kidneys, liver, spleen, lungs, stomach, and intestines were collected, weighed, and subjected to histopathological examination. This comprehensive approach aimed to evaluate the acute toxicity, mortality, hematological effects, and potential organ damage associated with the administration of ENZ-PLGA-PSAR-TPGS NPs.

##### 2.2.10.1 Pharmacokinetics

In accordance with the ethical approval (SPTM/2022/IAEC/18), nine male Albino Wistar rats weighing between 200 and 250 g were divided into three groups (n = 3) for proper conduct of pharmacokinetic experiments. The study aimed to compare the plasma concentration-time profiles of ENZ suspension and ENZ-PLGA-PSAR-TPGS NPs over a period of 48 h.

In the first group, rats were administered 15 mg/kg of ENZ intravenously, along with 0.9 % sodium chloride solution to enhance solubility. The second group received a 15 mg/kg dose of ENZ-PLGA-PSAR-TPGS NPs with 0.9 % w/v sodium chloride for improved solubility. Blood samples (500 μL) were collected from the rats' tail veins into heparinized tubes and cold centrifuged at 13,500 rpm for 10 min at 4 °C to separate plasma from blood. Subsequently, 100 μL of the separated plasma was mixed with 200 μL of acetonitrile, vortexed for 3 min to extract proteins, and cold centrifuged again at 13,500 rpm for 5 min at 4 °C.

*The supernatant was then diluted with methanol, passed through a membrane filter, and subjected to analysis using a High-Performance Liquid Chromatography (HPLC) system. Isocratic elution was performed with a mobile phase consisting of a* 20 mM *ammonium acetate buffer and acetonitrile in a 60:40 ratio, at a flow rate of 1.*0 mL/min*, utilizing a C18 column. This analytical approach allowed for the quantification of ENZ and facilitated the comparison of plasma concentration-time profiles between ENZ suspension and ENZ-PLGA-PSAR-TPGS NPs*.

##### 2.2.10.2 Tissue distribution

To assess the distribution and concentration of a medication within the body following delivery, tissue distribution studies were conducted. Twelve male albino Wistar rats were divided into two groups, each comprising six rats, following the approved institutional protocol by the Committee for the Purpose of Control and Supervision of Experiments on Animals (CPCSEA) (Ref. No.: SPTM/2022/IAEC/18).

The medications evaluated for tissue distribution were ENZ and the formulation ENZ-PLGA-PSAR-TPGS NPs. After administering the doses for 8 h, the rats were euthanized. Tissue samples, including liver, heart, kidney, lungs, brain, colon, small intestine, stomach, and rectum muscle tissues, were collected. Brain endothelial cells were obtained using a stereotaxic apparatus (USA, Model No. 51500). The collected tissue samples were homogenized in a cold physiological solution and filtered after rinsing with saline water (0.9 percent sodium chloride).

Subsequently, the samples were analyzed using RP-UHPLC (Dionex Ultimate, 3000) to determine the distribution and concentration of the medications in various tissues. This approach allowed for a comprehensive understanding of how ENZ and ENZ-PLGA-PSAR-TPGS NPs were distributed across different organs and tissues in the body [64].

#### 2.2.11 Stability

In the development of pharmaceutical preparations, ensuring stability is crucial to maintain the product's safety, efficacy, and quality throughout its shelf life. The approval and acceptance of pharmaceutical products are contingent upon meeting these criteria. Following the International Council for Harmonisation of Technical Requirements for Pharmaceuticals for Human Use (ICH) recommendations, a stability study was conducted on the produced polymeric nanoparticles (NPs) over a three-month period at three distinct storage temperatures: room temperature (25 ± 2 °C), higher temperature (40 ± 2 °C), and refrigerated conditions (2–8 °C). This systematic evaluation helps assess the potential impact of different storage conditions on the stability of the polymeric NPs, ensuring that the product remains suitable for use over its intended shelf life [65]. To assess the stability of the developed system, an evaluation was conducted by observing its physical characteristics, particle size, zeta potential, and Polydispersity Index (PDI). These parameters serve as key indicators of the system's stability over time. Monitoring changes in particle size, zeta potential, and PDI can provide insights into potential aggregation, precipitation, or other alterations in the system's physical properties. This comprehensive assessment helps ensure that the system maintains its intended characteristics and quality throughout the designated storage period, contributing to the overall stability and reliability of the pharmaceutical formulation.

#### 2.2.12 Statistical analysis

For the statistical analysis of the data, software tools including Microsoft Excel 2019, OriginPro 8.5, and GraphPad Prism 8 were employed. The data were presented as mean ± standard deviation (SD), and the entire experimental process was conducted in triplicate to ensure reliability and accuracy. To ascertain significant differences between groups, the student t-test was applied. For comparisons involving more than two groups, a one-way Analysis of Variance (ANOVA) was utilized. Statistical significance was defined at a P-value of 0.05, ensuring a rigorous evaluation of the obtained results.

## 3 Results and discussions

### 3.1 Spectroscopical analysis of ENZ

#### 3.1.1 Analysis by UV–visible spectroscopy

**Fig. 3:**
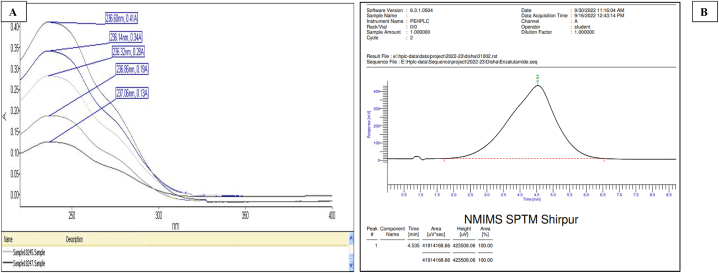
(A). Overlay display plot of Enzalutamide (ENZ) drug analyzed by spectroscopy method in UV–vis spectrophotometer. (B). Spectroscopical method of ENZ in HPLC.

Drug solution was scanned for maximum wavelength of absorbance at 236 nm as shown in Fig. 3A. The calibration curve from UV showed a R-squared value of 0.996 suggesting that the curve matches instrument data and equation of line is accurate and can be used for determination of unknown concentration.

### 3.2 Estimation of ENZ by RP-HPLC

It was done in order to confirm the purity of ENZ and detecting any kind of impurities present in them. Retention time was observed at 4.54 min as shown in Fig. 3B. The small peak observed at 1.0 min depicts the running of mobile phase in the column.

### 3.3 Box-Behnken design analysis and optimization

#### 3.3.1 Effect on particle size of ENZ-PLGA-PSAR-TPGS NPs

Among the formulations, DF14 showed the highest particle size at 212.2 ± 0.7 nm, while DF12 demonstrated the smallest particle size at 178.9 ± 2.3 nm **(refer to** Table 2**)**. The Polydispersity Index (PDI) values ranged from 0.1 to 0.2, indicating a relatively narrow size distribution. Notably, formulation DF14 displayed the highest PDI of 0.2, suggesting a broader range of particle sizes within this particular formulation. Conversely, formulation DF12 exhibited the smallest PDI of 0.1, signifying a more uniform particle size distribution.

**Table 2:** Effect of variation in independent variables on the characteristics of different polymeric NPs formulations (DOE).

Formulation code	PSAR concentration (%w/v)	PLGA concentration (%w/v)	Homogenization speed (rpm)	Particle size (nm)	Zeta Potential (mV)	Entrapment efficiency (%)
	X1	X2	X3	Y1	Y2	Y3
DF1	+1	0	0	206.2 ± 0.9	12.6 ± 0.8	68.2 ± 1.5
DF2	0	0	0	200.1 ± 2.3	11.1 ± 0.3	60.9 ± 0.2
DF3	0	0	0	199.1 ± 1.8	10.9 ± 1.7	59.1 ± 0.5
DF4	−1	0	−1	182.5 ± 1.2	8.6 ± 0.7	42.3 ± 1.6
DF5	0	0	0	196.9 ± 2.4	10.6 ± 0.4	54.6 ± 0.2
DF6	0	+1	+1	198.3 ± 1.2	10.5 ± 1.8	62.6 ± 0.3
DF7	+1	−1	0	204 ± 3.1	11.8 ± 0.8	64.8 ± 1.1
DF8	0	+1	−1	201.5 ± 4.5	11.4 ± 1.3	63.6 ± 1.5
DF9	−1	+1	0	194.8 ± 5.6	9.2 ± 0.9	48.3 ± 0.6
DF10	0	0	0	195.3 ± 4.2	9.8 ± 1.9	55.2 ± 2.3
DF11	0	0	0	197.3 ± 3.7	10.2 ± 0.2	58.3 ± 0.4
DF12	−1	0	+1	178.9 ± 2.3	8.4 ± 0.4	46.6 ± 1.8
DF13	0	0	+1	192.1 ± 1.7	9 ± 2.1	53.9 ± 1.2
DF14	+1	+1	0	212.8 ± 0.7	12.8 ± 1.8	75.4 ± 0.9
DF15	+1	0	+1	208.6 ± 1.6	13.1 ± 2.4	71.2 ± 0.7
DF16	−1	−1	0	179.5 ± 2.1	8.8 ± 1.5	40.3 ± 1.5
DF17	0	−1	−1	193.7 ± 2.4	9.1 ± 2.3	51.8 ± 1.9

Analysis of Variance (ANOVA) results **(refer to** Table 3**)** indicated a significant effect of factors such as the concentration of PLGA, PSAR, and homogenization speed on the particle size of these formulations. The quadratic expression of the design further indicated the effects on particle size, and the detailed results are provided below. Eq (1):

(Eq.1) Particle Size (Y1) = 197.40 + 12.12A + 4.63B- 1.25C- 1.750AB+ 0.5AC −0.5BCE −1.32A^2^ + 1.18B^2^ – 2.58C^2^

Were.

A: Concentration of Polysarcosine
B: Concentration of PLGA
C: Homogenization speed

**Table 3:** Summary of regression analysis for the measured responses.

Responses	Model	R^2^	Adj. R^2^	Predicted R^2^	S.D.	%CV	p-value	F-value
Particle size	Quadratic	0.970	0.932	0.698	2.48	1.26	0.0002	25.62
Zeta Potential	Quadratic	0.965	0.919	0.857	0.43	4.18	0.0003	21.42
%EE	Quadratic	0.974	0.953	0.921	2.13	3.74	<0.0001	37.03

Results of runs of DF2, DF3, DF5, DF10 and DF11 clearly indicated better reproducibility with coefficient of variance (%CV) of 1.26. P-value was of 0.0002 and F-value was of 25.62, which depicted significance of model on particle size. Correlation coefficient (R^2^) was of 0.97, depicting fitness presented data in model depicting comparison between the predicted values and experimental value. The F-value for lack of fit is 0.25, suggesting that the lack of fit is not significant when compared to pure error. In statistical terms, a lack of fit F-value is considered significant if it indicates a low likelihood of occurring due to noise that may obscure proper model fitting. In this case, the lack of fit F-value of 0.25 implies that there is a 25.69 % likelihood that any observed lack of fit is due to random noise rather than a deficiency in the model. A non-significant lack of fit is generally favorable, indicating that the model adequately represents the relationship between the variables and is not significantly impacted by random fluctuations or noise (Table 3).

The interpretation of statistical data shows increment in particle size w.r.t increment in concentration of polymers (X1 and X2) which was represented by positive coefficient in mentioned Eq. (1) whereas there is decrease in particle size when compared to increase in homogenization speed as indicated by negative coefficient in Eq. (1). Evaluation of interaction among all three independent variables as well indicated significant interactions happening among concentration of PLGA along with PSAR in formulations.

**Fig. 4:**
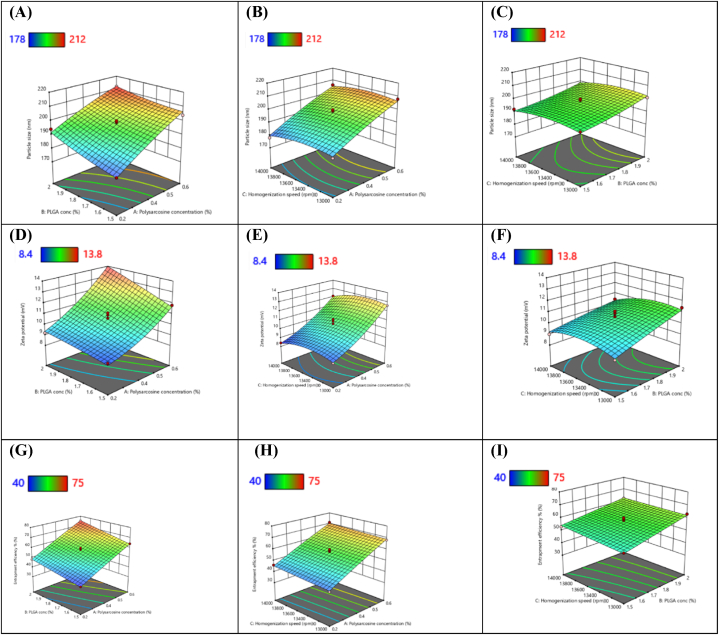
3D response surface plots indicating (A) the effect of two polymer concentrations on particle size, (B) the effect of the polymer and homogenization speed on particle size and (C) effect of PLGA polymer and homogenization speed on particle size. 3D response surface plots indicating (D) the effect of two polymer concentrations on zeta potential, (E) the effect of the polymer and homogenization speed on zeta potential and (F) effect of PLGA polymer and homogenization speed on zeta potential. 3D response surface plots indicating (G) the effect of two polymer concentrations on entrapment efficiency, (H) the effect of the polymer and homogenization speed on entrapment efficiency and (I) effect of PLGA polymer and homogenization speed on entrapment efficiency.

Fig. 4A shows that as the concentration of PSAR (X1) increases from 0.2 to 0.6 %w/v the particle size (Y1) is increasing from 178.9 ± 2.3 nm to 212.8 ± 0.7 nm. However, there is increase in particle size along with the change of concentration of PLGA (X2). This indicated significant increment in size of particle at higher concentrations which might be occurred because of increment in viscosity [66]. Firstly, there can be increment in viscosity value in accordance of decrement in evaporation rate of solvent utilized in organic solution while making polymeric NPs, which results into formation of larger size in particle [67]. Otherwise, viscosity at higher concentration of polymer results in creating resistance to breakdown droplets into particles of much smaller size and helps in decreasing causation of shear force which was produced by stirring while making ENZ-PLGA-PSAR-TPGS NPs [68]. Results of same were discovered included previous studies, which was analyzed by Stock's law. On basis of those differences in viscosity of medium creates resistance in collision of particles and might cause decrement in probability of breaking into smaller particle sizes [69].

Similarly, Fig. 4B shows decrease in particle size (Y1) from 212.8 nm to 179.8 nm as the homogenization speed (X3) increases from 13000 rpm to 14000 rpm. There is slightly more decrement seen in particle size after 135000 rpm speed of homogenization. It is suggested that shear force created by homogenizer in homogenization process leads to formation of small coacervates thus leading in reduction of particle size. Whereas similar pattern is observed in Fig. 4C where particle size is seen increasing with concentration of PLGA and decreasing in size of particle with increase in homogenization speed.

#### 3.3.2 Effect on zeta potential of ENZ-PLGA-PSAR-TPGS NPs

Zeta Potential of all 17 formulations were analyzed in order to assess caused effect of various dependent variables such as concentration of PSAR (X1) and PLGA (X2) and homogenization speed (X3). DF12 showed the lowest zeta potential that is 8.4 ± 0.4 mV and DF15 depicted highest zeta potential that is 13.1 ± 2.4 mV (Table 2).

ANOVA conducted results (Table 3) depicts much significant effect of independent variables such as concentration of PLGA, PSAR and homogenization speed on zeta potential of ENZ-PLGA-PSAR-TPGS NPs. Quadratic expression of model indicates effect on zeta potential and given mentioned Eq. (2):

(Eq.2) Zeta Potential (Y2) = 10.52 + 1.91A + 0.7625B −0.2250C + 0.4AB −0.0750AC −0.2250BCE +0.4150A^2^ -0.0350B^2^ - 0.5100C^2^

Where.

A: Concentration of Polysarcosine
B: Concentration of PLGA
C: Homogenization speed

Fig. 4D depicts that as concentration of PLGA increases, we can see there is slight decrease in zeta potential of the formulations whereas as the concentration of PSAR increases from 0.2 %w/v to 0.6 %w/v, there is a significant increase in zeta potential that is from 8.4 ± 0.4 mV to 13.1 ± 2.4 mV indicating significant effect on the stability of the nanoparticles. Eq. (2) also depicts the positive coefficient indicating increase of zeta potential in relation to increase in concentration of zeta potential whereas it shows negative coefficient in relation to concentration of PLGA.

Similarly, Fig. 4E depicts slightly negative or can be said as no effect of homogenization speed w.r.t zeta potential of all the formulations. As shown in above Eq. (2), we can conclude that −0.22 coefficient can be rounded off as 0 that is no correlation of homogenization speed w.r.t zeta potential of the ENZ-PLGA-PSAR-TPGS NPs formulation. PSAR has been proven to have a significant positive charge acting as a shield in formulation [70]. Observations are made similarly in Fig. 4F w.r.t null correlation with homogenization speed. The p-value of the quadratic model was 0.0003 that is significant as value is less than value of 0.05 and presented F-value of 21.42 depicts that model is significant and there is only 0.3 % chance of this value this large occurred due to mentioned noise. The R^2^ value that is 0.965 shows that the model is significant between the independent variable and dependent variables. The F-value for the lack of fit is seen as 0.28, indicates that lack of fit value is resulted as not significant in comparison to pure error. A significant resulted lack of Fit F-value has an 83.82 % likelihood of occurring because of noise that obscures proper model fitting (Table 3).

#### 3.3.3 Effect on drug entrapment efficiency of ENZ-PLGA-PSAR-TPGS NPs

Drug entrapment of all formulations was analyzed in order to assess causative effect of various variables like concentration (%w/v) of PSAR and %w/v of PLGA and homogenization speed on entrapment of the drug ENZ at constant content. DF16 showed the lowest drug entrapment efficiency that is 40.3 ± 1.5 % and DF14 depicted highest drug entrapment efficiency that is 75.4 ± 0.9 % (Table 2).P-value represented as <0.0001 and F value represented as 37.03 depicted quite significance of applied model on size of particle. R^2^ value of 0.984 as shown in table depicted fitness of given data in the model showing comparison between the predicted values and experimental data. The F-value for the lack of fit, 0.25, indicates that lack of fit is not significant in nature when comparison to pure error. A significant Lack of Fit F-value has an 85.88 % likelihood of occurring because of noise that obscures proper model fitting (Table 3).

Quadratic expression of design indicates causative effect on %Entrapment efficiency and given mentioned Eq. (3):

(Eq.3) % Entrapment Efficiency (Y3) = 57.20 + 12.75A + 5.06B–1.00C + 0.75AB −0.25AC −0.75BCE +0.475A^2^ + 0.0250B^2^ + 0.0250C^2^

Where.

A: Concentration of Polysarcosine
B: Concentration of PLGA
C: Homogenization speed

As shown in Fig. 4G, it can be interpreted that as the concentration of PLGA (X1) and PSAR (X2) increases, the drug entrapment efficiency (Y3) also increases. Even though, concentration of PSAR depicts slightly more effect on the entrapment efficiency as compared to concentration of PLGA. It was found that higher the concentration of polymer, the higher it would provide space to encapsulate drug and produce relatively complex matrix [71].

Similarly, as shown in Fig. 4H, it depicts that there is no effect seen in entrapment efficiency w.r.t homogenization speed. It can be clearly depicted that as homogenization speed is increasing from 13000 rpm to 14000 rpm, there is constant graph seen in the entrapment efficiency but there is significant increase in increase in entrapment efficiency from 40.3 % to 75.4 % as concentration of PSAR increases. Fig. 4I depicts constant increment w.r.t PSAR and homogenization speed. has been proven to increase better entrapment efficiency due to its better solubility.

### 3.4 Optimization

Optimization of independent variables were analyzed through desirability approach. Numeric optimization was achieved by setting various constraints and analysing optimal levels of independent variables (X1, X2 and X3), which help in maximizing desired response variables (Y1, Y2 and Y3). Independent batches of optimized formulations were prepared three times by utilizing optimal constraints of independent variables mentioned by Design-Expert® software. Predicted and actual values of particle size (Y1), zeta potential (Y2) and drug entrapment efficiency (Y3) are mentioned in Table 4. By analysing data, actual values of these variables were found to be in compliance along with predicted ones. This created a close agreement amongst predicted and actual values and helped in depicting reliability of optimization process for formulation of Polymeric nanoparticles scaffolded with ENZ [72] and DF15 is considered as optimized formulation.

**Table 4:** Predicted and actual values of dependent variables for the optimized formulation.

Concentration of PSAR (%w/v)	Concentration of PLGA (%w/v)	Homogenization speed (rpm)	Particle size (nm)	Zeta Potential (mV)	Entrapment efficiency (%)	Desirability
0.6	1.75	14,000	Actual:208.6 Predicted:206.3	Actual: 13.11 Predicted: 12.61	Actual: 71.2 Predicted: 70.25	0.939

### 3.5 Morphology of optimized ENZ-PLGA-PSAR-TPGS NPs

**Fig. 5:**
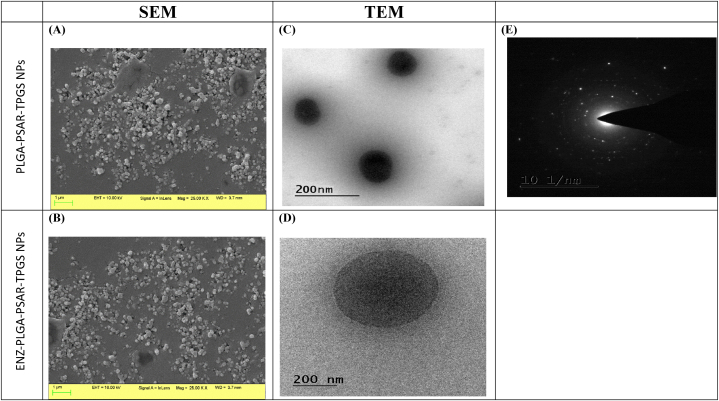
FESEM images of (A) PLGA-PSAR-TPGS NPs (Placebo), (B) ENZ-PLGA-PSAR-TPGS NPs (optimized formulation) and TEM images (C) PLGA-PSAR-TPGS NPs (Placebo), (D) ENZ-PLGA-PSAR-TPGS NPs (optimized formulation); (E) SAED of ENZ-PLGA-PSAR-TPGS NPs demonstrating ring pattern by TEM.

FESEM was conducted to analyze surface form and surface morphology of optimized formulation and image depicted a spherical shape along with smoothness on surface. As demonstrated in Fig. 5B and **A**, SEM depicts the surface morphology of optimized ENZ-PLGA-PSAR-TPGS NPs as well as PLGA-PSAR-TPGS NPs respectively. The nanometric size indicates that the particle size is appropriate and wouldn't pass Reticuloendothelial system leading to better bioavaibility. The particle size measurement from Malvern is a little of greater significance than the one from SEM. This can be explained by pointing out that differential light scattering determines the hydrodynamic size of a nanoparticle, which is size of NP along with liquid layer surrounding it, whereas SEM measures actual size of NPs.

Whereas, TEM was conducted to analyze morphology of optimized ENZ-PLGA-PSAR-TPGS NPs. TEM imaging as shown in Fig. 5C and **D** depicted particle spherical in shape with appropriate size. It was observed small bright damp-coloured spherical particles inside the polymer material which might be core of PLGA and PSAR. It also depicted uniform distribution of the bright coloured material in encapsulated NP matrix. The nanoparticle size was significant in size w.r.t SEM and Malvern particle size results. Selected Area electron diffraction (SAED) can be observed in Fig. 5E depicting the low intensity of polymeric ring results in poor crystallization of the nanoparticle and thus can be concluded that it is amorphous which might ensure proper encapsulation of the drug ENZ inside the polymeric material [73].

The TEM images of the polymeric nanoparticles (NPs) revealed distinct and unique particle sizes that closely matched the findings of the dynamic light scattering (DLS) technique. DLS was utilized to measure the solvent layer adhering to the surface of the particles, known as the hydrodynamic particle size, which was consistently found to be larger than the real particle size as determined by TEM.

Moreover, the electron diffraction pattern in the figure indicated the amorphous nature of the nanoparticles, as evidenced by the diffused ring structure. This observation was consistent with the results obtained from differential scanning calorimetry (DSC), Fourier transform infrared spectroscopy (FTIR), and X-ray diffraction (XRD).

The observed surface morphology of the prepared nanoparticles in the TEM images indicated the expected spherical and non-aggregated particles. In the projected TEM images, the coating of PLGA, polysarcosine, and TPGS was clearly evident. This research showcases the use of transmission electron microscopy (TEM) for studying the loading of drugs onto polymeric nanoparticles, providing valuable insights into their size and structure. These findings contribute to the advancement of drug delivery systems.

### 3.6 Drug release studies

**Fig. 6:**
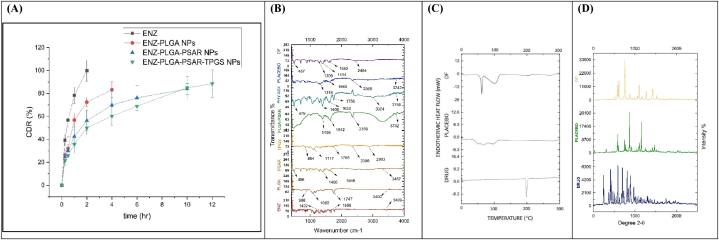
A. in vitro drug release of optimized ENZ-PLGA-PSAR-TPGS NPs along with drug ENZ, ENZ-PLGA NPs, ENZ-PLGA-PSAR NPs. B. FT-IR spectra of ENZ, PLGA, PSAR, TPGS, PLGA-PSAR NPs, Physical Mix, Placebo (PLGA-PSAR-TPGS NPs) and DF (ENZ-PLGA-PSAR-TPGS NPs) C. DSC of ENZ, Placebo (PLGA-PSAR-TPGS NPs) and DF (ENZ-PLGA-PSAR-TPGS NPs) D. XRD pattern of ENZ, Placebo (PLGA-PSAR-TPGS NPs) and DF (ENZ-PLGA-PSAR-TPGS NPs).

*In vitro* drug release from ENZ, ENZ-PLGA NPs, ENZ-PLGA-PSAR NPs, ENZ-PLGA-PSAR-TPGS NPs were performed in PBS at physiological conditions maintaining 37 °C and pH 5.5 by utilizing dialysis tube process, in order to predict the in vivo behaviour. PBS pH 5.5 buffer was considered due to acidic nature of ENZ having pKa value of 4.16 and PSAR's tendency to swell and release drugs in acidic environment present in tumour [74]. The observed pattern of release in optimized ENZ-PLGA-PSAR-TPGS NPs had a higher and sustained release,88.5 ± 22.5 % in 12 h, compared to pure drug which had poor pattern of release and ENZ-PLGA NPs having 83.2 ± 15.34 % in 4 h and ENZ-PLGA-PSAR NPs having release of 83.2 ± 23.4 % in 10 h. From Fig. 6A, The analysis suggests that the polymeric nanoparticles (NPs) exhibited a biphasic release behavior, characterized by an initial burst release followed by a controlled release over an extended period. The initial burst release can be attributed to the drug that was adsorbed on the surface of the polymeric NPs, as well as the drug that was encapsulated near the outer surface of the nanoparticles. This rapid release phase may involve the drug molecules present on or near the surface of the NPs being readily available for release into the surrounding environment. Following the initial burst release, the controlled release phase suggests a more sustained and controlled liberation of the encapsulated drug over an extended timeframe. This behavior is desirable in drug delivery systems, as it allows for an immediate therapeutic effect followed by a prolonged release, potentially improving the overall efficacy and duration of the treatment. The biphasic release pattern is often tailored to meet specific therapeutic requirements and optimize the therapeutic outcome of the drug delivery system [75]. while later sustained release maybe attributed by polymers present in the nanoparticle. R^2^ obtained was 0.91 which describes good correlation between formulations and proving ENZ-PLGA-PSAR-TPGS NPs as suitable sustained release pattern.

### 3.7 Drug release kinetics

Different types of kinetic models like zero order, first order, Higuchi and Korsmeyer-Peppas models were utilized in order to explain order and mechanism of release of drug. R^2^ was used to identify most appropriate fit model to explain drug releasing behaviour from NPs. Value of R^2^ of different models are mentioned respectively in Table 5. It was discovered from ENZ releasing from Polymeric NPs follow Higuchi model (R^2^ = 0.9888) release which shows a diffusion-controlled release. Bayesian information criterion (BIC), Akaike information criterion (AIC), and root-mean-square deviation (RMSE) were statistical metrics for evaluating a model's fit to data. They assist us in comparing models and selecting the most appropriate one for the data. Utilizing RMSE, drug release kinetics models can be assessed. A lower RMSE indicates that the model and the data are more accurately matched. A statistical model's fit to a dataset is assessed using AIC and BIC. For quality of fit and complexity, they penalise models with extra parameters. To compare models and select one with a good fit and parsimony, one can utilise AIC and BIC. The best measure value is determined by the context and analytical goals. The resulting R^2^ values for the First order and Korsmeyer-Peppas model for ENZ-PLGA-PSAR-TPGS NPs were less than 0.9, suggesting that the ENZ's release patterns did not comply to these models. In contrast, R^2^ values for the Zero order, Higuchi models were more than 0.90 (Table 5). The diffusional constant (n) present in Korsmeyer-Peppas equation was utilized to predict mechanism of ENZ drug release from drug delivery system (if n < 0.5 then Fickian diffusion, n = 0.5–1.0 then non-Fickian transport). Here, n < 0.5, that is in this result value of 0.457 describes release of drug ENZ form PLGA-PSAR-TPGS matrix of NPs following Fickian diffusion mechanism [[76], [78]].

**Table 5:** Drug release kinetics depicting various models.

Model	R^2^	K	RMSE	AIC	BIC	Drug Release exponent “n”
Zero order	0.9169	2.68	2.34	3.31	3.45	–
First order	0.7945	4.11	4.23	7.31	8.32	–
Higuchi	0.9883	1.74	1.56	2.37	3.23	–
Korsmeyer-Peppas	0.794	4.11	4.23	7.31	8.32	0.457

### 3.8 FTIR studies

The ATR-FTIR studies conducted in this analysis provide a reliable method for assessing the compatibility between all structural components present in the formulation, including Enzalutamide (ENZ), Poly Lactic-*co*-Glycolic Acid (PLGA), polysarcosine (PSAR), d-α-Tocopheryl polyethylene glycol 1000 succinate (TPGS), PLGA-PSAR, the physical mixture, PLGA-PSAR-TPGS NPs (Placebo), and ENZ-PLGA-PSAR-TPGS NPs (DF).

Characteristic bands in the FTIR spectra confirm the presence of specific functional groups. For instance, in ENZ, bands at 3439 cm-1, 1659 cm-1, and 1422 cm-1 correspond to (-N-H-), (–NH–CO–NH–) groups, and (-CH2CO-) group, respectively. The PLGA spectrum exhibits peaks at 1747 cm-1 (C=O) and 998 cm-1 (OH end group), while polysarcosine displays peaks at 1649 cm-1 (secondary amine) and 3457 cm-1 (primary and secondary amine). TPGS peaks at 3738 cm-1 (terminal hydroxyl group) match with the results of Raju A. et al.

No significant appearance or disappearance of peaks is observed in the optimized formulation (DF) and placebo with respect to drug ENZ and individual polymers and emulsifiers. This absence of changes in peak patterns suggests no chemical interaction between the drug, polymers, and other excipients used in the formulation.

Compatibility is further analyzed between the drug and other polymers using the FTIR peak matching method. For example, peaks at 864 cm-1 in TPGS FTIR (alkene bending) are seen at 889 cm-1 in the Drug Formulation (DF). Similarly, the peak at 1642 cm-1 indicating monosubstituted C=C alkene in PLGA-PSAR formulation matches with the peak at 1642 cm-1 in DF. Peaks of PLGA and PSAR are observed at low intensity in the placebo and DF spectra, confirming proper encapsulation (Fig. 6B).

Overall, the FT-IR peak matching method indicates the successful incorporation of ENZ into the polymeric NP matrix without any significant chemical reactions occurring between the polymers and the drug.

### 3.9 Differential scanning calorimetry studies

The drug differential scanning calorimetry (DSC) study serves as a crucial tool to comprehend the crystalline behavior, thermodynamic properties, and melting point of Enzalutamide (ENZ), placebo, and the optimized polymeric nanoparticles. The DSC spectra of ENZ, PLGA-PSAR-TPGS NPs, and ENZ-PLGA-PSAR-TPGS NPs are depicted in Fig. 6C.

In the DSC analysis, ENZ exhibited an endothermic melting peak at 201 °C. The presence of this peak signifies the temperature at which the drug undergoes a phase transition from a solid to a liquid state. Importantly, the DSC analysis of ENZ encapsulated in polymeric nanoparticles showed no interaction with the polymers, indicating the absence of any drug-polymer interactions. This lack of interaction is a positive outcome, as it suggests that the encapsulation process did not induce any chemical changes in the drug.

However, it is noted that the endothermic peak of TPGS, which typically occurs at 40 °C, was shifted to 50 °C in the presence of ENZ-PLGA-PSAR-TPGS NPs. This shift in peak intensity may be attributed to the encapsulation of ENZ within the PLGA-PSAR-TPGS nanoparticles, causing a modification in the thermal behavior of TPGS. The shift in peak temperature suggests an alteration in the thermodynamic properties, potentially influenced by the interaction between TPGS and the polymeric matrix containing ENZ.

Additionally, ENZ characteristic peak vanished from the DSC spectra of ENZ-PLGA-PSAR-TPGS NPs, demonstrating complete encapsulation of ENZ within the nanoparticles. Surprisingly, the DSC spectra of PLGA-PSAR-TPGS NPs displayed distinctive peaks at 50 °C, 70 °C, and 103 °C. The melting point of PLGA was discovered by T. Musumeci et al., in 2006 while constructing sustained release colloidal suspension of Docetaxel. Differential scanning calorimetry analysis (DSC) reported that DTX was molecularly dispersed in polymeric matrices. Similar results were reported in our investigation, where the strength of PLGA endothermic peak was found to be lowered and the melting endotherm moved from 60 °C to 65 °C in ENZ-PLGA-PSAR-TPGS NPs formulation, showing encapsulation of ENZ-PLGA nanoparticles with PSAR and TPGS [77].

### 3.10 XRD studies

The characteristic diffraction angle peaks (2ϴ) observed at 243°, 413°, 592°, 698°, and 815° for pure Enzalutamide (ENZ) drug indicated the crystalline nature of the molecule. These peaks exhibited various patterns and variable intensities. However, in the X-ray diffraction (XRD) patterns of the polymeric nanoparticles, no strong peaks were evident.

Upon lyophilization of ENZ-PLGA-PSAR-TPGS NPs, the typical peaks disappeared, and a shift occurred at 613° with low intensity. This shift indicated that the nanoparticles had transitioned from a crystalline to an amorphous state (refer to Fig. 6D). The reduction in the crystalline nature of ENZ-PLGA-PSAR-TPGS NPs has implications for their solubility in water. Amorphous forms are generally more soluble than crystalline forms, which may enhance the release of drugs from the polymeric matrix through diffusion.

The lack of crystallinity in ENZ-encapsulated polymeric nanoparticles aligns with the observed in vitro drug release kinetics. The diffraction peaks of ENZ-scaffolded polymeric NPs suggest the formation of a polymeric complex, indicating a transition from a crystalline to an amorphous form. This transformation in the structural state of the nanoparticles can influence their dissolution behavior and release kinetics, potentially enhancing the therapeutic effectiveness of the drug delivery system.

### 3.11 in vitro anticancer studies\

#### 3.11.1 SRB assay

**Fig. 7:**
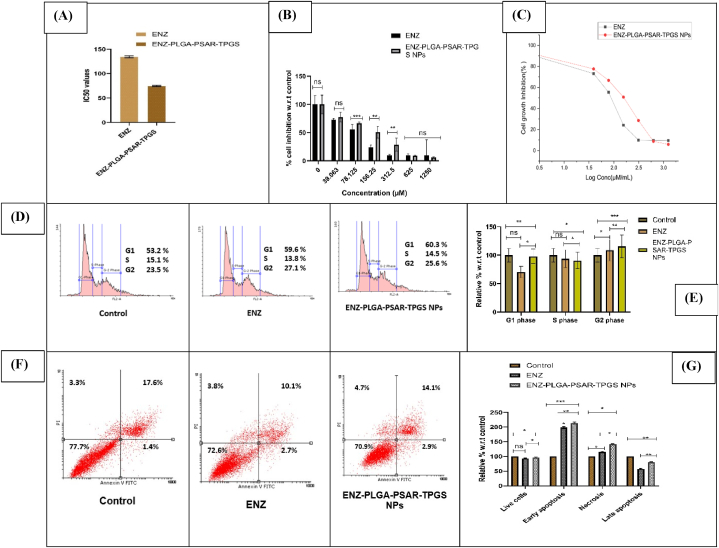
A. IC50 values of drug ENZ and ENZ-PLGA-PSAR-TPGS NPs. B. cell inhibition percentage w.r.t control at different concentrations (0,39,78.1, 156.2, 312.5, 625,1250 μM). C. cell inhibition comparison of ENZ and optimized ENZ-PLGA-PSAR-TPGS NPs using p-test (values are represented as mean ± SD (n = 3)) at 0.05 significance indicating (p > 0.05: ns; p < 0.05: *; p < 0.01: **; p < 0.001: ***). D. Cell cycle analysis of control, ENZ and ENZ-PLGA-PSAR-TPGS NPs. E. Graphical representation of the cells present in G1, S and G2 phase using Dunet's One way ANOVA test (values are represented as mean ± SD (n = 3)) at 0.05 significance indicating (p > 0.05: ns; p < 0.05: *; p < 0.01: **; p < 0.001: ***). F. Scatter graphical representation of cellular apoptosis in Control, ENZ, ENZ-PLGA-PSAR-TPGS NPs. G. Cell inhibition comparison of ENZ and optimized ENZ-PLGA-PSAR-TPGS NPs at different stages of cellular apoptosis such as Early apoptosis, Live cells, Late apoptosis and necrosis using Bonferroni's One way ANOVA test (values are represented as mean ± SD (n = 3)) at 0.05 significance indicating (p > 0.05:ns; p < 0.05:*; p < 0.01:**; p < 0.001:***).

The IC50 values for Enzalutamide (ENZ) and ENZ-PLGA-PSAR-TPGS NPs were determined to be 134.5 μM and 74.58 μM, respectively, after a 24-h incubation period in the HCT 116 cell line (refer to Fig. 7A). The lower IC50 value for ENZ-PLGA-PSAR-TPGS NPs compared to ENZ alone suggests an enhanced cytotoxic effect of the nanoparticle formulation. This enhancement can be attributed to the sustained release of ENZ and the increased internalization of nanoparticles facilitated by the adhesion of TPGS. The effectiveness of TPGS in coating the nanoparticles was confirmed, contributing to the multifunctionality of the nanoparticles and potentially reducing side effects in chemotherapy. In the experimental setup, ENZ was considered the control group, and the efficacy of ENZ-PLGA-PSAR-TPGS NPs in inducing cytotoxicity was reported. Notably, PLGA-PEG-NPs showed nonsignificant results in cytotoxicity against the HCT 116 cell line, indicating minimal anticancer effects of the existing PLGA and PEG in the formulation. The primary role of TPGS in this experiment was to enhance target specificity. Although marginal changes in the rate of growth inhibition (%) were observed in ENZ-PLGA-PSAR-TPGS NPs treatment, indicating a higher cell growth rate at a specific concentration, overall, the coated nanoparticles demonstrated positive changes in the cytotoxicity of HCT 116 cancer cell lines (refer to Fig. 7B). Furthermore, it's worth noting that, at the initial phase and in all treatments, HCT116 cells were viable at 100 %. However, at the concentration of 39.06 μM with different formulations, cells did not exhibit any inhibitory response, and their growth was exponential. The growth rate of HCT116 cell lines demonstrated inhibitory effects starting from the treatment with 74.58 μg/mL of ENZ-PLGA-PSAR-TPGS NPs (refer to Fig. 7C). The percentage of cell inhibition, compared to non-treated control cells of HCT116 cell lines, showed a significant effect of polymeric NPs compared to ENZ drug at low concentrations.

The findings from this study suggest that polymeric NPs, particularly those coated with TPGS, exhibit a significant anticancer effect on HCT 116 cell lines, potentially providing a more effective and targeted therapeutic approach for colorectal cancer [79]. establishing results with the present SRB assay of ENZ-PLGA-PSAR-TPGS NPs.

#### 3.11.2 Cell cycle analysis

Antitumor agents can induce apoptosis by activating signalling pathways, leading to G2/M phase arrest. Flow cytometry is used to measure cell growth in different cell cycle phases (G1, S, and G2/M). ENZ and ENZ-PLGA-PSAR-TPGS NPs were chosen for further analysis of their effects on cell cycle progression in the HCT116 cell line. Briefly, HCT116 cells were incubated with these compounds at their IC50 concentration for 24 h. There was an inhibitory effect on the percentage of apoptotic cells, as indicated by an increase in cells at G1 phase at 60.3 % when compared with ENZ at 49.6 %. In contrast to that, ENZ-PLGA-PSAR-TPGS NPs at G2/M phase show 13.8 % inhibitory effect as compared to ENZ that is 14.5 %. The percentage of HCT116 cells treated with ENZ-PLGA-PSAR-TPGS NPs in S and G0/G1 phases showed increase at 27.1 % when compared to drug ENZ at 25.6 % causing cycle arrest (Fig. 7D). Fig. 7E depicts the comparison of ENZ and ENZ-PLGA-PSAR-TPGS NPs affected on the HCT116 cell lines relative percentage w.r.t G1 phase, S phase and G2 phase. Jiang Biying et al. explained relationship of UBA2 in A549 cell line, which depicted increment in G1 and G2/M phase while decrement in S phase cells having p-value lesser than 0.005 depicting the best fit of the model [80]. These results clearly indicated that ENZ-PLGA-PSAR-TPGS NPs show apoptotic effect bit higher than pure drug ENZ at G1 phase of the cell cycle.

#### 3.11.3 Cellular apoptosis assay

ENZ-PLGA-PSAR-TPGS NPs and ENZ induces apoptosis in HCT116 cells (Fig. 7F). Single-cell suspensions were prepared from tumour tissues of control and treated groups as described in materials and methods. Apoptotic cells were determined by FITC Annexin-V staining and flow cytometry. The presented scatter plots shown in Fig. 7F consist of four quadrants; the upper left depicts necrotic cells (PI positive); upper right quadrant shows late apoptotic cells where PI is negative and Annexin V is positive; lower right quadrants depict early apoptosis and lower left quadrant shows live cells. The flow cytometric analyses are shown as histograms and the percentage of apoptotic cells are shown as bar graph. Control group; ENZ group and ENZ-PLGA-PSAR-TPGS NPs treated HCT116 cells. Statistical analyses were performed with One way ANOVA Dunet's test *p < 0.01 compared with control group. It's been observed that at bottom left quadrant there is presence of live cells which are 72.6 % present in ENZ treated HCT-116 cell lines whereas it is 70.9 % of live cells present in ENZ-PLGA-PSAR-TPGS NPs treated cell line leading to decrease in number of live cells as of the lower IC50 concentration as compared to drug ENZ. Early-stage apoptosis can be denoted as initial stage of apoptosis which leads to activation of caspases cleaving cellular components present in the cell. 2.7 % of early apoptosis has been observed at ENZ-treated cell whereas decrement is seen at 2.9 % of cells treated with ENZ-PLGA-PSAR-TPGSNPs. Late apoptosis of ENZ is seen as 10.1 % whereas its 14.1 % in ENZ-PLGA-PSAR-TPGS NPs depicting the higher irreversible apoptosis along with loose membrane permeability with lower toxic concentration as compared to ENZ and necrosis is also increased in cells treated with ENZ-PLGA-PSAR-TPGSNPs when compared to ENZ. Similarly, Wang C. et al. wanted to analyze the IncRNA UCA1 function promoting inhibitory effect on gastric cancer cells. They performed MTT assay and apoptosis assay to see the viability of cancerous cells w.r.t IncRNA. They found reduced viability of the tumorous cells and increased apoptosis in UCA1 knockdown cells [81]. Graph in Fig. 7G similarly depicts relative percentage w.r.t control at different stage of cell apoptosis.

#### 3.11.4 Reactive oxygen Spieces assay (ROS)

**Fig. 8:**
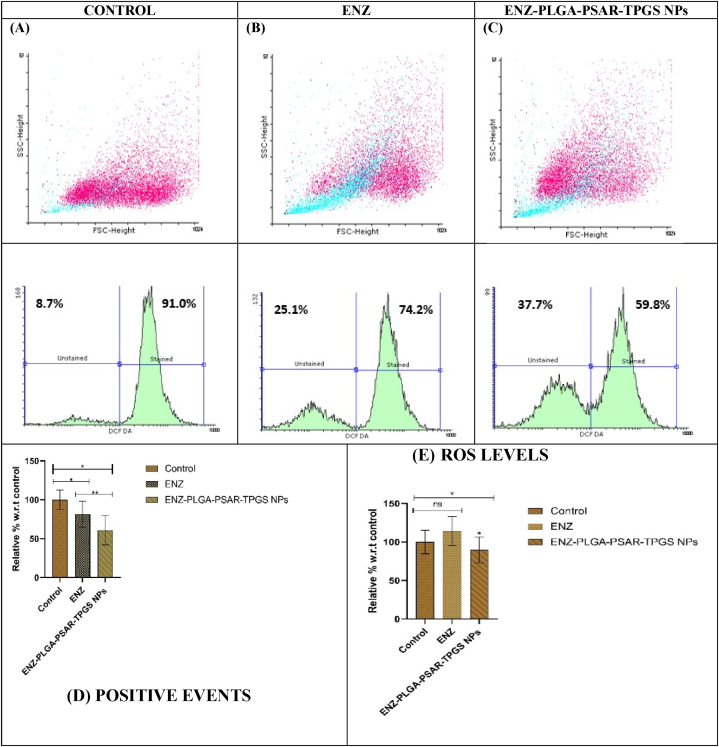
Graphical representation of ROS estimation in (A) Control; (B) ENZ; (C) ENZ-PLGA-PSAR-TPGS NPs; (D) and (E) ROS positive % cells and presence of ROS levels in comparison with control, ENZ and ENZ-PLGA-PSAR-TPGS NPs using Dunet's One way ANOVA test (values are represented as mean ± SD (n = 3)) at 0.05 significance indicating (p > 0.05: ns; p < 0.05:*; p < 0.01:**; p < 0.001:***).

ROS assay depicts highly reactive molecules which are generated during metabolic mechanism in cells which are in higher concentration and causes damage to cells or tissues with their oxidation process. There is higher value of ROS seen in cancerous cells due to increased metabolic activity in higher number of cells due to cancer. The role of ROS in cancer cell survival and death is bidirectional. The cancer cells undergo apoptosis because of oxidative stress caused by decrease in ROS. When compared to ENZ (Fig. 8A–C), HCT-116 cancer cell lines treated with ENZ-PLGA-PSAR-TPGS NPs indicate lower formation of ROS. ENZ-PLGA-PSAR-TPGS NPs treatment for HCT-116 cancer for 24 h may have had comparable results. Fig. 8D depicts the %ROS positive cells and Fig. 8E depicts the ROS levels with ENZ treated HCT-116 cells line and ENZ-PLGA-PSAR-TPGS NPs treated HCT-116 cell lines. They are performed in triplicate manner and can be depicted as decrease of ROS in ENZ-PLGA-PSAR-TPGS NPs w.r.t drug ENZ. Sivandzade F. et al. conducted studies on mitochondrial membrane potential data analysis related to the effects of xenobiotics, such as tobacco smoking, on blood-brain barrier endothelial cells, encompassing both mouse primary (mBMEC) and a mouse-based endothelial cell line (bEnd 3) in a side-by-side comparison using JC-1 dye giving fluorescence activity. Results In response to both TS and e-Cig exposure, data demonstrated a decreased red (590 nm) to green (529 nm) fluorescence intensity ratio in connection to mitochondrial depolarization, which is clearly visible in the fluorescence images. The outcomes also demonstrated that e-Cig had a less negative effect than TS. Information is offered as a useful illustration of how JC-1 dye can be used to measure changes in both qualitative and quantitative ways [82].

#### 3.11.5 Mitochondrial membrane potential

**Fig. 9:**
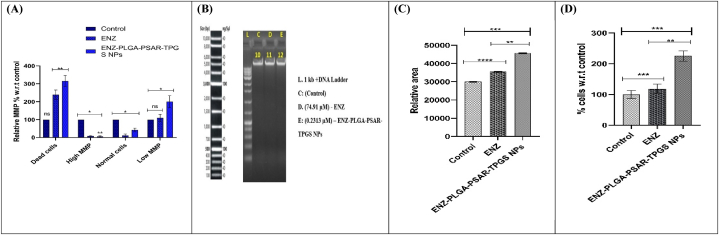
A. Graphical representation of mitochondrial membrane potential estimation between Control, ENZ, ENZ-PLGA-PSAR-TPGS NPs using Dunet's One way ANOVA test (values are represented as mean ± SD (n = 3)) at 0.05 significance indicating (p > 0.05:ns; p < 0.05:*; p < 0.01:**; p < 0.001:***). B. DNA fragmentation assay mentioning DNA ladder along with control, ENZ, ENZ-PLGA-PSAR-TPGS NPs. C. Relative area of cells w.r.t control, ENZ, ENZ-PLGA-PSAR-TPGS NPs using Dunet's One way ANOVA test (values are represented as mean ± SD (n = 3)) at 0.05 significance indicating (p > 0.05: ns; p < 0.05: *; p < 0.01: **; p < 0.001: ***). D. Cleavage in percent of cells w.r.t control, ENZ, ENZ-PLGA-PSAR-TPGS NPs using Dunet's One way ANOVA test (values are represented as mean ± SD (n = 3)) at 0.05 significance indicating (p > 0.05: ns; p < 0.05: *; p < 0.01: **; p < 0.001: ***).

Mitochondrial membrane potential is estimated by the electrochemical potential needed to pump out the H^+^ ions while the conversion from ADP to ATP. Mitochondria is the only organelle which exhibits the significant membrane potential. The energy level is low denoting that apoptosis/dead process in progress i.e. the cell is dying. Using flow cytometry, cells labelled with the JC-1 dye were examined. A decline in the red/green fluorescence intensity ratio is a sign of a drop in the potential of the mitochondrial membrane. Low MMP can be determined by the red to green ratio whereas High MMP can be detected with green to red fluorescence intensity. The JC-1 dye enters and concentrates in the activated, negatively charged mitochondria and spontaneously produces red luminous J-aggregates in healthy cells with normal cell. In contrast, the JC-1 dye also reaches the mitochondria in sick or apoptotic cells, although to a lower extent since the interior of the mitochondria is less negative due to increased membrane permeability and the ensuing loss of electrical potential [83]. After a 24-h incubation period, Fig. 9A demonstrates that ENZ and ENZ-PLGA-PSAR-TPGS significantly depolarized the mitochondrial membrane of the cancer cells because of larger % of low MMP cells. Higher the high mitochondrial membrane potential it denotes good functioning of the cancer cell and it is observed in Fig. 9A that ENZ-PLGA-PSAR-TPGS NPs contain low number of high MMP cells. ENZ treated HCT-116 cell lines have 52.7 % cells with 41.7 % of low MMP cells whereas, it can be observed that ENZ-PLGA-PSAR-TPGS NPs have 30.3 % live cells with 65.3 % low MMP cells depicting better efficacy of Polymeric nanoparticle. High MMP was 3.5 % in ENZ, which is slightly low in compared to 3.8 % of ENZ-PLGA-PSAR-TPGS NPs treated cell (Fig. 9A).

Fauzi A. et al. analyzed the effect of Tualang honey on human breast cancer (MCF-7 and MDA-MB-231 cell line) for 24 h at different concentrations. There is clear depiction of increment in membrane potential showing the significant effect of honey on HeLa cells moving from upper right quadrant High MMP cells to left upper quadrant dead/necrosis cells showing Capase-3/7 activation [84]. These results depict that MMP of ENZ-PLGA-PSAR-TPGS NPs is lower than ENZ treated cells in treating colon cancer.

#### 3.11.6 DNA fragmentation assay

It is conducted in order to extract DNA from cells or tissues and then conducting electrophoresis separating fragments of DNA and is kept in comparison with the DNA ladder made with 10,000 base pairs of DNAS (Fig. 9B). It is mainly used to analyze the toxicity of the drug formulation w.r.t drug ENZ and its efficiency with cellular processes related to apoptosis. There are 5000 base pairs present in control, 0.2313 μM of ENZ-PLGA-PSAR-TPGS NPs and 74.91 μM of ENZ indicating lower concentration of ENZ-PLGA-PSAR-TPGS NPs in comparison to ENZ as shown in Fig. 9B. Fig. 9C depicts Relative area and Fig. 9D depicts cell cleavage in number of cells respectively with control, ENZ and ENZ-PLGA-PSAR-TPGS NPs, indicating that prepared formulation shows more inhibition as compared to drug ENZ depicting that ENZ-PLGA-PSAR-TPGS NPs are less toxic as compared to the drug was more effective in cleaving of DNA leading to apoptosis of CRC cells.

Barani M. et al. conducted DNA fragmentation on Thymoquinone niosomes and Carum Carvil extract niosomes. DNA ladder was prepared with 10,000 base pairs of DNA and it could be seen that there was no apoptotic activity seen in Carvil niosomes whereas there was apoptotic activity seen in Thymoquinone niosomes via DNA fragmentation assay [85].

#### 3.11.7 Wound healing assay

HCT-116 cell lines are monolayer cultured and a scratch is done in the cell layer where they are treated with ENZ and ENZ-PLGA-PSAR-TPGS NPs and their closure of the wound is measured with a period of time as if the drug possess anti-cancer activity, it might inhibit the cell line growth causing avoidance of the wound to heal. The in vitro scratch assay is a valuable method for studying the effects of anticancer drugs on the migration and wound healing ability of cancer cells. The results of this assay support the hypothesis that anticancer drugs, such as 10.13039/501100001550ENZ and ENZ-PLGA-PSAR-TPGS NPs, inhibit the spread and migration of cancer cells. Tumor angiogenesis, the formation of new blood vessels to support tumor growth, involves processes such as cell migration, which can be assessed using scratch assays.

In the study, HCT-116 cancer cells were treated with ENZ and ENZ-PLGA-PSAR-TPGS NPs for 24 and 48 h. The analysis aimed to determine non-toxic concentrations of the treatments while assessing their impact on cell migration. The results indicated that ENZ inhibited the wound area after 48 h compared to the control, and a higher inhibition was observed in the area of HCT-116 cells treated with ENZ-PLGA-PSAR-TPGS NPs.

**Fig. 10:**
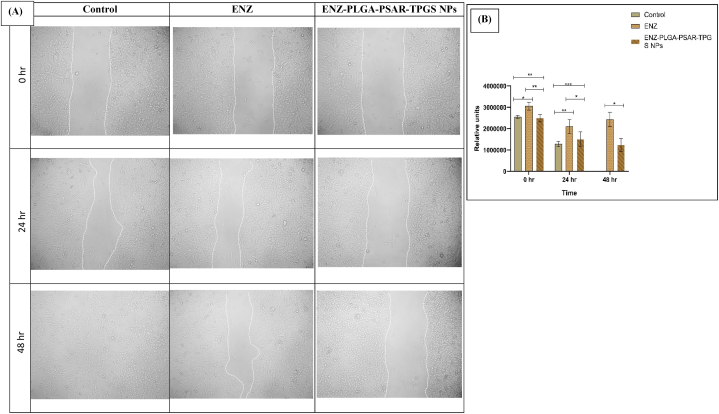
A. High contrast microscopic images of erythrocytes during haemolysis (%) studies after 100 μg/mL of ENZ, PLGA-PSAR-TPGS NPs (Placebo) and ENZ-PLGA-PSAR-TPGS NPs with saline treatment (negative controlled) and Triton X-100 treatment (positive controlled) during the 0.5th and 10th hours. B. Amount of LDH release after treating ENZ and ENZ-PLGA-PSAR-TPGS NPs at different time intervals. The error bars in this figure represent the standard error of the mean, calculated from three independent experiments. The bars show the range within which the true mean is likely to lie with 95 % confidence.

The use of crystal violet staining at 48 h helped determine the effect of the treatments on HCT-116 cell viability. Lower concentrations of ENZ-PLGA-PSAR-TPGS NPs (0.2313 μM) and ENZ (74.91 μM) were used for wound healing, aiming for low toxicity while maintaining a strong inhibitory effect at lower concentrations (refer to Fig. 10A).

To compare treated cells with controls, the control cells were set at 100 %, and the viability inhibition of treated cells was expressed as a percentage (refer to Fig. 10B). The scratch-wound assay provides insights into fundamental aspects of cell migration, including velocity, tenacity, and directional preference.

The images taken at the 48-h mark following the scratch-wound assay clearly indicate that both ENZ and ENZ-PLGA-PSAR-TPGS NPs have anticancer effects by inhibiting the motility of HCT-116 cells. The assay demonstrates the ability of these treatments to impede wound healing and migration, supporting their potential as effective anticancer agents.

Comparatively, Alejandra et al. conducted a similar in vitro scratch assay using the HaCat cell line, where they created horizontal wounds and analyzed wound healing gaps with an optimized plugin for ImageJ. Their study focused on automated identification of wound healing size, correcting average wound width, and quantifying parameters such as area, wound area fraction, average wound width, and width deviation. Both studies highlight the versatility and significance of scratch assays in assessing the impact of drugs on cell migration and wound healing processes [86]. Our results are consistent with those of the aforementioned study, lending integrity to our investigation.

### 3.12 *In vivo* studies

#### 3.12.1 Haemolysis studies

**Fig. 11:**
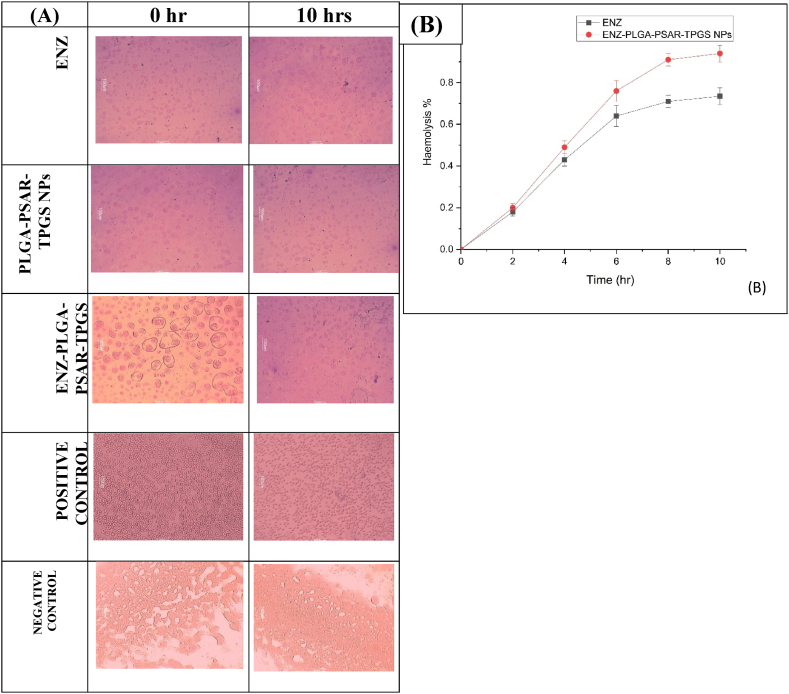
A. High contrast microscopic images of erythrocytes during haemolysis (%) studies after 100 μg/mL of ENZ, PLGA-PSAR-TPGS NPs (Placebo) and ENZ-PLGA-PSAR-TPGS NPs with saline treatment (negative controlled) and Triton X-100 treatment (positive controlled) during the 0.5th and 10th hours. B. Amount of LDH release after treating ENZ and ENZ-PLGA-PSAR-TPGS NPs at different time intervals. The error bars in this figure represent the standard error of the mean, calculated from three independent experiments. The bars show the range within which the true mean is likely to lie with 95 % confidence.

*In vivo* toxicity study was considered to be most crucial for carrying out in order to analyze nano formulation behaviour along with blood. Fig. 11A depicted profile of percentage haemolysis absorbance with various hour changes of erythrocytes along with 100 μg/ml of treatment containing drug and polymeric NP formulation and results as shown in Fig. 11B showed ENZ-PLGA-PSAR-TPGS NPs roughly showed no haemolysis rate even at maximum amount of NPs reached 0.95 % which is in the category of non-haemolytic biomaterials [87]. ENZ drug and PLGA-PSAR-TPGS NPs can be qualitatively compared in Fig. 11A and can be depicted as of no occurrence of eruption or lysis of the RBCs w.r.t to negative control formulation containing Triton X causing the lysation process of the cell. The drug ENZ and ENZ-PLGA-PSAR-TPGS NPs formulation depicted appropriate blood compatibility thus, leading to a suitable formulation for intravenous administration.

#### 3.12.2 Platelet aggregation

**Fig. 12:**
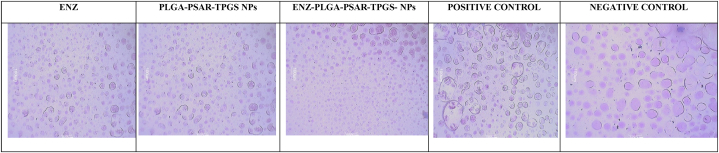
Platelet aggregation of Leishman-stained glass slides with Control, ENZ, PLGA-PSAR-TPGS NPs (Placebo), ENZ-PLGA-PSAR-TPGS NPs with saline treatment (negative controlled) and Triton X-100 treatment (positive controlled).

Platelet aggregation studies are used to carry out the compatibility of the formulation w.r.t bleeding and thrombosis characterization which can be directly affected on platelet function. The black representation on the Leishman-stained slides show the platelets with no aggregation in ENZ and ENZ-PLGA-PSAR-TPGS NPs (Fig. 12). Platelet count was conducted with help of counting denoting no changes in aggregation of platelet of ENZ and ENZ-PLGA-PSAR-TPGS NPs along with its safe use for administration.

#### 3.12.3 Acute toxicity studies

**Fig. 13:**
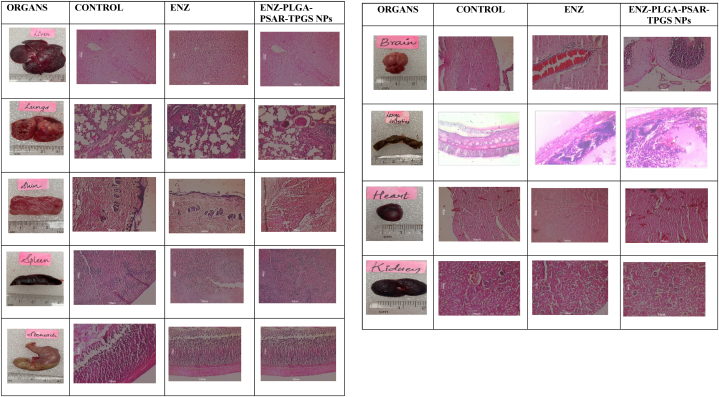
Histopathology slides of different organs that is Brain, Colon, Heart, kidney, liver, lungs, skin, spleen and stomach after being administered ENZ and ENZ-PLGA-PSAR-TPGS NPs w.r.t Control. Histopathology slides of different organs that is Brain, Colon, Heart, kidney, liver, lungs, skin, spleen and stomach after being administered ENZ and ENZ-PLGA-PSAR-TPGS NPs w.r.t Control.

Acute toxicity studies can be used to assess the safety of the carrier by looking at a variety of factors, including food and water intake, body weight, sickness symptoms, fever, diarrhoea, behavioural patterns, and haematological and histological analysis. Physical examination revealed no symptoms of disease, and water-food consumption kept in normal condition over 14 days in both control-test groups, proving proposed system's compatibility. After collecting blood samples in heparinized tubes on fifteenth day to avoid blood clotting, several haematological tests were conducted. According to Table 6A, the haematological analyses were normal and comparable in both the control and test groups, demonstrating compatibility of created polymeric NP system. To assess toxicity of created carrier system within essential organs, the histopathological observation was also conducted. All animals were sacrificed for this purpose on the fifteenth day, and important vital organs namely liver, heart, lungs, spleen, kidney, stomach and intestine were removed and weighed; Table 6B shows that neither the control group nor the test group had any discernible differences. Numerous different slides made from vital organs were examined histopathologically with the help of an optical microscope, and as shown in Fig. 13, There are no indications of toxicity, lesions, distortion, or deformation. ENZ-PLGA-PSAR-TPGS NPs are safe in nature, efficient drug delivery vehicles for possible delivery of chemotherapeutic drug, according to the results of the acute toxicity investigation.

**Table 6A:** Haematological parameters of the control group and Test group (ENZ-PLGA-PSAR-TPGS NPs).

Haematology	Group I (Control)	Group II (Test)
Haemoglobin (Hb) (g/dL)	16.6 ± 0.65	12.6 ± 0.4
Red blood cells × 10^6^ mm^3^	6.0 ± 0.79	6.2 ± 0.74
White blood cells × 10^9/^ L	7.2 ± 1.2	7.82 ± 1.6
Platelets × 10^9^/L	4.22 ± 0.32	4.86 ± 0.73
Lymphocytes (%)	59.52 ± 2.2	57.82 ± 3.2
Monocytes (%)	1.72 ± 1.9	1.68 ± 2.2
Neutrophils (%)	46.5 ± 1.44	44.2 ± 2.6
Mean corpuscular volume (%)	69.52 ± 3.14	71.5 ± 2.8
Mean corpuscular haemoglobin (pg/cells)	23.5 ± 0.67	22.4 ± 0.8
Mean corpuscular haemoglobin concentration (%)	32.06 ± 5.56	30.5 ± 11.6

**Table 6B:** Organ weight (gm) of Group-I (control) and Group-II (test) containing ENZ-PLGA-PSAR-TPGS NPs.

Haematology	Group I (Control)	Group II (Test)
Haemoglobin (Hb) (g/dL)	16.6 ± 0.65	12.6 ± 0.4
Red blood cells × 10^6^ mm^3^	6.0 ± 0.79	6.2 ± 0.74
White blood cells × 10^9/^ L	7.2 ± 1.2	7.82 ± 1.6
Platelets × 10^9^/L	4.22 ± 0.32	4.86 ± 0.73
Lymphocytes (%)	59.52 ± 2.2	57.82 ± 3.2
Monocytes (%)	1.72 ± 1.9	1.68 ± 2.2
Neutrophils (%)	46.5 ± 1.44	44.2 ± 2.6
Mean corpuscular volume (%)	69.52 ± 3.14	71.5 ± 2.8
Mean corpuscular haemoglobin (pg/cells)	23.5 ± 0.67	22.4 ± 0.8
Mean corpuscular haemoglobin concentration (%)	32.06 ± 5.56	30.5 ± 11.6

#### 3.12.4 Pharmacokinetic studies

The administration of Enzalutamide-loaded Poly Lactic-*co*-Glycolic Acid (PLGA) nanoparticles coated with polysarcosine and d-α-Tocopheryl polyethylene glycol 1000 succinate (TPGS) (ENZ-PLGA-PSAR-TPGS NPs) to all rat groups resulted in an increase in various pharmacokinetic parameters compared to free Enzalutamide (ENZ). The observed parameters included the initial concentration of the drug in plasma, volume of distribution, biological half-life, area under the curve (AUC), and mean residence time (MRT). In contrast, the clearance value was significantly decreased.

These findings indicate that ENZ-PLGA-PSAR-TPGS NPs exhibit extensive tissue binding after intravenous administration, suggesting their potential as a tool for improving systemic availability, prolonging systematic distribution, and extending the half-life of the ENZ drug. The mean residence time (MRT) of ENZ-PLGA-PSAR-TPGS NPs was notably longer (40.56 ± 6.74) than that of ENZ alone, suggesting that ENZ-PLGA-PSAR-TPGS NPs could prevent opsonin protein adhesion. This extended MRT may be attributed to the stealthy nature of the nanoparticles, allowing them to evade the reticuloendothelial system and thus prolong systemic circulation (Table 7).

**Table 7:** Pharmacokinetic parameters of ENZ and optimized ENZ-PLGA-PSAR-TPGS NPs after administering in rats intravenously.

Parameters	ENZ	ENZ-PLGA-PSAR-TPGS NPs
C (ng/mL)	226.24 ± 31.2	400.249 ± 22.56
V_d_ (mL)	1400.45 ± 3.56	1125.67 ± 5.43
T_1/2_ (hr^−1^)	4.2 ± 1.18	38.4 ± 4.52
Cl (mL/hr)	90.45±	45 ± 6.34
AUC (ng.mL/hr)	1497 ± 80.46	2056 ± 125.6
MRT (h)	5.34 ± 2.43	40.56 ± 6.74

Statistical analysis using Dunnett's student t-test confirmed that ENZ-PLGA-PSAR-TPGS NPs had significantly higher maximum plasma drug concentrations (Cmax), total clearance (CL), and AUC compared to free ENZ. These pharmacokinetic parameters collectively suggest the potential of ENZ-PLGA-PSAR-TPGS NPs for enhanced therapeutic efficacy and sustained drug release.

#### 3.12.5 Tissue distribution studies

In the pharmacokinetic and biodistribution studies conducted according to the approved protocol (SPTM/2022/IAEC/18), 12 rats were sacrificed 8 h after administration. Tissue samples, including liver, heart, colon, small intestine, stomach, brain, rectum muscle, and kidney, were collected for analysis. The use of a stereotaxic device (New Standard™ Stereotaxic Instrument, Model no.: 51500, USA) facilitated the collection of undamaged brain endothelium cells. Tissue samples were cleaned with saline water (0.9 percent sodium chloride), dried with filter paper, homogenized in a cold physiological solution, and the filtered extracted solution was analyzed using RP-UHPLC (Dionex Ultimate, 3000).

Results from Bonferroni's multiple comparison test revealed that the distribution of ENZ-PLGA-PSAR-TPGS NPs in heart, colon, and rectum tissues was significantly higher compared to ENZ alone. This suggests that ENZ-PLGA-PSAR-TPGS NPs have a higher distribution through the bloodstream, with efficient resistance in the stomach. 10.13039/100014337Furthermore, the distribution of nanoparticles in the small intestine was significantly higher (p < 0.05), supporting the concept of rapid clearance established in pharmacokinetic studies. The lower drug deposition in the brain indicates reduced absorption of polymeric NPs through the Blood-Brain Barrier (BBB), suggesting less neurotoxicity. The reduced liver distribution also implies a minimized rate of biodistribution of ENZ-PLGA-PSAR-TPGS NPs. The characteristic property of ENZ and its active metabolite N-desmethyl ENZ being metabolized in the liver, especially by CYP2C8 and CYP3A4, is reflected in the low concentration in the liver, indicating that ENZ-PLGA-PSAR-TPGS NPs help bypass liver metabolism. Additionally, the ENZ molecule entrapped inside PLGA-PSAR is not readily available for metabolism, further delaying biotransformation.

The comprehensive review of biodistribution results indicates that rectum tissue has a higher distribution of both ENZ and ENZ-PLGA-PSAR-TPGS NPs (Table 8). Therefore, ENZ-PLGA-PSAR-TPGS NPs show potential as a useful tool for colorectal targeting of ENZ, presenting a promising approach for the treatment of colorectal cancer (CRC).

**Table 8:** Tissue distribution of ENZ and ENZ-PLGA-PSAR-TPGS NPs in different organs.

Organs	Concentration of ENZ (ng/mL)	Concentration of ENZ-PLGA-PSAR-TPGS NPs (ng/mL)
Heart	0.8 ± 1.102	1.1 ± 0.210
Liver	1.2 ± 0.130	0.9 ± 0.103
Lungs	0.5 ± 0.102	1.2 ± 0.1028
Spleen	0.3 ± 0.1	0.45 ± 0.203
Kidney	0.57 ± 0.21	0.65 ± 0.06
Stomach	0.21 ± 0.12	0.38 ± 0.209
Brain	0.7 ± 0.10	0.79 ± 0.32
Colon	0.95 ± 0.5	1.21 ± 0.07
Rectum	3.07 ± 0.4	3.67 ± 0.689

### 3.13 Stability studies

The stability of the generated ENZ-PLGA-PSAR-TPGS NPs was assessed by storing them for three months at three different temperatures: 2–8 °C in the refrigerator, 25 ± 2 °C at room temperature, and 40 ± 2 °C at higher temperature. The results indicate that there was no significant difference in particle size and zeta potential after three months of storage of ENZ-PLGA-PSAR-TPGS NPs at the refrigerator temperature of 2–8 °C (p > 0.05). Furthermore, minimal differences were observed after three months of storage at both room temperature and elevated temperature. These findings suggest that the ENZ-PLGA-PSAR-TPGS NPs Table 9 demonstrated stability during the three-month storage period under various temperature conditions.

**Table 9:** Stability data of ENZ-PLGA-PSAR-TPGS NPs after 3 months stored at different temperatures.

Formulation code	Storage conditions	Time	Particle size (nm)	Zeta Potential (mV)	PDI
F15	Initial	3 months	208.6 ± 1.6	13.11 ± 0.14	0.238 ± 0.02
F15	2–8 °C	3 months	209.5 ± 1.02	12.65 ± 0.12	0.245 ± 0.04
F15	25 ± 2 °C	3 months	211.6 ± 0.9	11.34 ± 1.5	0.285 ± 0.12
F15	40 ± 2 °C	3 months	217 ± 0.6	11.01 ± 1.3	0.270 ± 0.36

## 4 Potential limitation and future direction

In the current investigation, the fabrication of Enzalutamide (ENZ)-loaded Poly Lactic-*co*-Glycolic Acid (PLGA) nanoparticles coated with polysarcosine and d-α-Tocopheryl polyethylene glycol 1000 succinate (TPGS) demonstrates a significant step forward in colorectal cancer therapy. The careful optimization of fabrication parameters, supported by advanced characterization techniques, underscores the potential of these nanoparticles for sustained drug release and targeted delivery. Despite the compelling outcomes in in vitro and in vivo assessments, several avenues for future exploration emerge. Comprehensive long-term toxicity studies are imperative to ascertain the safety profile of the nanoparticles. Further investigations into pharmacokinetics, biodistribution, and scalability will fortify the translational potential. Exploring responsive features in the nanoparticles and their adaptability to dynamic cancer microenvironments presents an exciting avenue. In general, this study sets the stage for a promising colorectal cancer treatment strategy, with identified areas for refinement and future research to enhance its clinical applicability.

## 5 Conclusion

The ENZ-loaded polymeric NPs were fabricated appropriately and evaluated for various spectroscopical, physicochemical, in vitro characterization assays and in vivo characterization studies. Variables i.e., particle size, zeta potential and drug entrapment efficiency were optimized via response surface methodology through box Behnken approach. ANOVA analysis demonstrated effect of polymers improving solubility of ENZ (which is BC class II) and helps in target specification. Optimized formulation founded nanosized particle with good mono dispersion and well sustained, Fickian diffusion drug release profile. FT-IR depicted perfect compatibility of polymers and drug. DSC and XRD suggested proper valuation of drug coated in polymer. In vitro cyto-toxicity studies depicted significant effect w.r.t low concentration of ENZ-PLGA-PSAR-TPGS NPs as compared to plain drug ENZ indicating that formulated polymeric NPs are safe and less cyto-toxic in nature with better bioavaibility. In vivo studies showed no haemolysis and no platelet aggregation upon administration in rats with ENZ and ENZ-PLGA-PSAR-TPGS NPs proving its biocompatibility with blood. Acute toxicity studies depicted no lesions, distortions, food or water intake capability or any change in weight of organs making it safe for use. Pharmacokinetics and tissue distribution studies depicted lesser clearance time from the body and showed low concentration of ENZ-PLGA-PSAR-TPGS NPs in liver as compared to ENZ indicating CYP inhibition. Stability of ENZ-PLGA-PSAR-TPGS NPs was found to be appropriate after 3 months of storage in different conditions. Thus, ENZ-PLGA-PSAR-TPGS NPs are discovered to be a significant novel approach for treating CRC as compared to drug ENZ.

## Funding statement

The project did not received any fundings.

## Ethics statement

The Iin-vivo animal studies were approved by The Institutional Animal Ethics Committee (IAEC) with reference number SPTM/2022/IAEC/18.

## Data availability statement

The data would be made available on request.

## CRediT authorship contribution statement

**Disha Shah:** Writing – review & editing, Writing – original draft, Software, Resources, Project administration, Methodology, Formal analysis, Data curation, Conceptualization. **Sankha Bhattacharya:** Writing – review & editing, Writing – original draft, Visualization, Validation, Supervision, Software, Methodology, Investigation, Formal analysis, Data curation, Conceptualization. **Girdhari Lal Gupta:** Validation, Methodology, Formal analysis, Data curation. **Ketan Hatware:** Visualization, Validation, Methodology, Investigation. **Arinjay Jain:** Writing – original draft, Visualization, Supervision, Investigation. **Laxmi Manthalkar:** Writing – original draft, Visualization, Validation, Methodology, Investigation. **Niraj Phatak:** Visualization, Validation, Resources, Methodology, Investigation. **Putrevu Sreelaya:** Writing – original draft, Visualization, Validation, Resources, Methodology.

## Declaration of competing interest

The authors declare the following financial interests/personal relationships which may be considered as potential competing interests:Sankha Bhattacharya reports financial support, administrative support, and equipment, drugs, or supplies were provided by NMIMS School of Pharmacy & Technology Management. Sankha Bhattacharya reports a relationship with NMIMS School of Pharmacy & Technology Management that includes: employment. Sankha Bhattacharya has patent pending to Not Applicable. NA If there are other authors, they declare that they have no known competing financial interests or personal relationships that could have appeared to influence the work reported in this paper.

## Abbreviations

ENZ: Enzalutamide
PLGA: Poly lactic co-glycolic acid
PSar: Polysarcosine
TPGS: Tocopherol polyethylene glycol succinate
FESEM: Field emission scanning electron microscopy
TEM: Transmission electron microscopy
NP: Nanoparticles
CRC: Colorectal cancer
AR: Androgen receptor
CRPC: Castration Resistant protein cancer
MTT: 3-(4,5-dimethylthiazol-2-yl)-2,5-diphenyl tetrazolium bromide
MDR: Multi-drug resistance
PVA: Polyvinyl alcohol
PBS: Phosphate buffer solution
FT-IR: Fourier transform Infrared spectroscopy
DSC: Differential scanning calorimetry
XRD: X-ray diffraction
SRB: Sulforhodamine B assay
PI: Propidium iodide
DMEM: Dulbecco's Modified Eagle's Medium
OECD: Organisation for Economic Co-operation and Development
ICH: International Council for Harmonisation
SD: Standard deviation
ANOVA: Analysis of Variance
DLS: Dynamic light scattering
SAED: Scanning area of electron diffraction
AIC: Akaike Information criterion
BIC: Bayesian information criterion
RMSE: Root mean squared deviation
DF: drug formulation a.k.a ENZ-PLGA-PSAR-TPGS NPs
DTX: Docetaxel
MMP: Mitochondrial membrane potential
ROS: Reactive oxygen species
ADP: Adenosine phosphate
ATP: Adenosine tri-phosphate
DNA: Deoxyribonucleic acid
JC-1 dye: fluorescent cationic carbocyanine dye
MRT: Mean residence time
