# Supplementary material for: d-α-tocopheryl polyethylene glycol 1000 succinate surface scaffold polysarcosine based polymeric nanoparticles of enzalutamide for the treatment of colorectal cancer: In vitro, in vivo characterizations
Source: Heliyon. 2024 Feb 2;10(3):e25172. doi: 10.1016/j.heliyon.2024.e25172 (PMC10850913; doi:10.1016/j.heliyon.2024.e25172)
Supplement: Multimedia component 1 [file mmc1.docx]

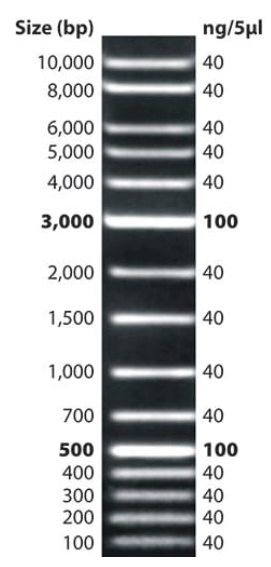

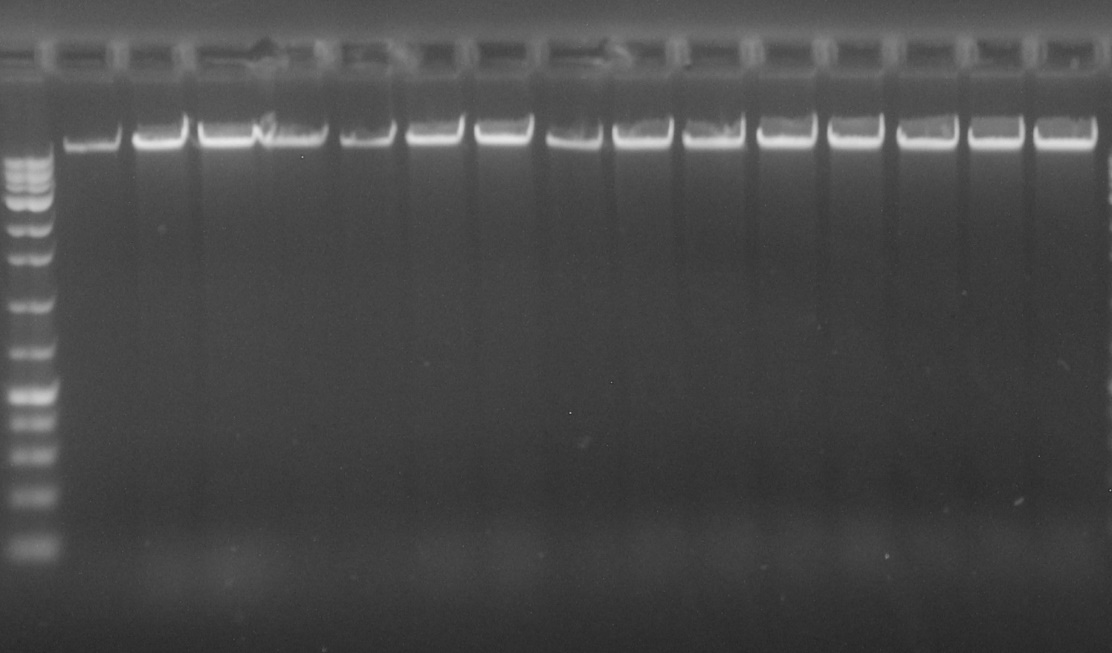


**Western blot-Fig.9B**

**L. 1 kb +DNA Ladder**

**1. (Control) 2. (0.6742 µM)- S1, 3. (13.61 µM)**

**4. (Control) 5. (17.52 µM) - S2 6. (1.053 µM)**

**7. (Control) 8. (1.526 µM) – S3, 9. (90.74 µM)**

**10. (Control) 11. (0.2313 µM) – S4 12. (74.91 µM)**

**13. (Control) 14. (3.109 µM) – S5 15. (104.2 µM)**
